# Systematic review: pain, cognition, and cardioprotection—unpacking oxytocin’s contributions in a sport context

**DOI:** 10.3389/fphys.2024.1393497

**Published:** 2024-06-10

**Authors:** Péter Szabó, Sara Bonet, Roland Hetényi, Dániel Hanna, Zsófia Kovács, Gyöngyvér Prisztóka, Zuzana Križalkovičová, József Szentpéteri

**Affiliations:** ^1^ Faculty of Sciences, Institute of Sports Science and Physical Education, University of Pécs, Pécs, Hungary; ^2^ Faculty of Humanities, University of Pécs, Pécs, Hungary; ^3^ Medical School, Institute of Transdisciplinary Discoveries, University of Pécs, Pécs, Hungary; ^4^ Faculty of Medicine Osijek, Josip Juraj Strossmayer University of Osijek, Osijek, Croatia; ^5^ RoLink Biotechnology Kft., Pécs, Hungary; ^6^ Hungarian National Blood Transfusion Service, Budapest, Hungary; ^7^ Szentágothai Research Centre, University of Pécs, Pécs, Hungary; ^8^ National Virology Laboratory, University of Pécs, Pécs, Hungary; ^9^ Faculty of Health Sciences, Institute of Physiotherapy and Sport Science, Department of Sport Science, Pécs, Hungary

**Keywords:** cardioprotection, cognition, exercise, measurement, oxytocin, pain

## Abstract

**Introduction:**

This systematic review investigates the interplay between oxytocin and exercise; in terms of analgesic, anti-inflammatory, pro-regenerative, and cardioprotective effects. Furthermore, by analyzing measurement methods, we aim to improve measurement validity and reliability.

**Methods:**

Utilizing PRISMA, GRADE, and MECIR protocols, we examined five databases with a modified SPIDER search. Including studies on healthy participants, published within the last 20 years, based on keywords “oxytocin,” “exercise” and “measurement,” 690 studies were retrieved initially (455 unique records). After excluding studies of clinically identifiable diseases, and unpublished and reproduction-focused studies, 175 studies qualified for the narrative cross-thematic and structural analysis.

**Results:**

The analysis resulted in five categories showing the reciprocal impact of oxytocin and exercise: Exercise (50), Physiology (63), Environment (27), Social Context (65), and Stress (49). Exercise-induced oxytocin could promote tissue regeneration, with 32 studies showing its analgesic and anti-inflammatory effects, while 14 studies discussed memory and cognition. Furthermore, empathy-associated *OXTR* rs53576 polymorphism might influence team sports performance. Since dietary habits and substance abuse can impact oxytocin secretion too, combining self-report tests and repeated salivary measurements may help achieve precision.

**Discussion:**

Oxytocin’s effect on fear extinction and social cognition might generate strategies for mental training, and technical, and tactical development in sports. Exercise-induced oxytocin can affect the amount of stress experienced by athletes, and their response to it. However, oxytocin levels could depend on the type of sport in means of contact level, exercise intensity, and duration. The influence of oxytocin on athletes’ performance and recovery could have been exploited due to its short half-life. Examining oxytocin’s complex interactions with exercise paves the way for future research and application in sports science, psychology, and medical disciplines.

**Systematic Review Registration:**

https://www.crd.york.ac.uk/prospero/display_record.php?RecordID=512184, identifier CRD42024512184

## 1 Introduction

Technology has made it increasingly difficult for children to interact and physically play with their peers. Movement and time spent together, when they gather in physical education classrooms, are clearly of paramount importance (Knaus et al., 2020). Through oxytocin secretion, sports might present opportunity to combat diseases, aggression, and bullying at school. Empathy and social care, related to oxytocin, may help this process (Jolliffe and Farrington, 2011). Finding the most appropriate sport is therefore key.

A brief overview of the relationship between oxytocin and exercise further warrants this review, as not much is explicitly known about the interplay between them. We do not know what the best sport is to aid the secretion of this hormone. This could be answered if a precise, stable, and robust measuring methodology, that could be followed in sports context, was available. The cardioprotective effect provided by exercise is already well established in sports and medical literature (Sakamoto et al., 2011). However, a deeper understanding of this relationship could provide a great benefit for sports science.

Oxytocin is a nonapeptide produced by neurohypophysis, most well-known for its role in social bonding, trust, love and intimacy, empathy, childbirth, and lactation. First identified in 1906 by Sir Henry Dale, who observed its ability to cause uterine contractions, it was later found to have much more complex functions and effects on both autonomic and cognitive processes. It is known today that oxytocin receptors are not only present in the central nervous system (amygdala, hippocampus, prefrontal cortex, and ventral tegmental area) but in other tissues as well—the uterus, heart, gastrointestinal tract, etc., Still, oxytocin’s role might be underestimated in the sports science field. Strikingly, this gap in the literature has neither been emphasized nor explored at the time of writing this review. In humans, oxytocin increases both sympathetic and parasympathetic cardiac activity (Brailoiu et al., 2013), the understanding of which would yield valuable insight for health sciences. Even though progressive efforts are made (Michelini, 2007a; Michelini, 2007b) animal studies document a more detailed description of the relationship between oxytocin, cardioprotection, hypertension, and exercise than current research on humans. For the afore mentioned reasons we believe that there is a strong need for the investigation of oxytocin’s exercise-related benefits.

In Wistar Kyoto (WKY) and Spontaneously Hypertensive (SHR) rats, walking and running on a treadmill decreased resting heart rate due to oxytocin (Michelini, 2007a; Michelini, 2007b; Ceroni et al., 2009; Higa-Taniguchi et al., 2009; Cavalleri et al., 2011). Exercise increases oxytocin, consequently boosting neuroplasticity, aiding its role in cardiac functions, and resting heart rate even in aging Wistar (W) rats using treadmills (Michelini, 2007a; Michelini, 2007b; Santos et al., 2018; Rocha-Santos et al., 2020). For cardioprotection, pre-treating, healing cardiac tissue, and hypertension-related issues oxytocin also shows positive and promising results in WKY, SHR, Sprague Dawley (SD), and W rats after either exercise-related or exogenous oxytocin adage (Martins et al., 2005; Moghimian et al., 2012; Gutkowska et al., 2016; Wang et al., 2020).

Looking at stress responses and recovery times, oxytocin might be a useful asset in both animals and humans (Stanić et al., 2017; Gümüs et al., 2015). Oxytocin’s role in muscle mass and metabolic health is not yet fully understood (Stanić et al., 2017). However, even intranasal oxytocin may enhance muscle mass and improve metabolic profiles (Espinoza et al., 2021). This could be crucial for sports and exercise programs aimed at older or sedentary individuals. In addition, moderate-intensity exercise and oxytocin displayed hepatoprotection in comparison with other treatments, believed to be attached to its antioxidant and anti-inflammatory effects (ELKady et al., 2021).

Aerobic involuntary exercise was able to induce significant oxytocin changes in rodents. Swimming training seems to be a powerful tool to elicit exercise-induced oxytocin change usually applied through the Morris Water Maze (MWM). However, the result of such stimuli connected to physical stress seems to be outcome-dependent and possibly connected to behavioral despair which is important data for treating depression (Porsolt et al., 2001; Peijie et al., 2003; Engelmann et al., 2006). Exercise-induced oxytocin may also help to avoid drug self-administration behaviors in W and SD rats (Farzinpour et al., 2019; Carson et al., 2010). This phenomenon may be explained by the anti-stress and anti-depressant effect of oxytocin, which is not yet fully understood (Crestani et al., 2013). Unsurprisingly, oxytocin release can be induced by vigorous aerobic exercise like swimming training in C57BL6 mice and likely also in humans (Kim and Han, 2016; Azari et al., 2023). The interaction of hope, positive and negative reinforcement, and oxytocin could possibly hold staggering discoveries in the future as previously tested in a well-known study on rats where hope meant survival and increased mobility (Castagné et al., 2011). Furthermore, exogenous or administered oxytocin is capable of increasing exercise performance in SHR and W rats, which, if tested in humans, could lead to improved sports performance and recovery (Cruz et al., 2013; Juif and Poisbeau, 2013; Liu and Xue, 2017; Shima et al., 2022). It is also possible that higher oxytocin levels can be associated with better trainability in horses (Kim et al., 2021; Kim et al., 2023) and dogs (Mitsui et al., 2011), changing their proclivity to react preferably to ambiguous stimuli. Plasma oxytocin was also slightly elevated by aerobic running exercises in dogs, pulling a sled together (Leggieri et al., 2019). Of course, for now, the meaning of these findings is unclear for human physiology.

Voluntary exercise, however, was not always able to stimulate oxytocin enough to elicit significant change. In male W, Lewis, SHR, and SD rats voluntary wheel running did not alter oxytocin significantly, moreover, the response again seemed stress-dependent (Bakos et al., 2007; Bakos et al., 2008; Takahashi et al., 2022). Elevated oxytocin levels offer cardioprotection through vasodilation and regulation of the resting heart rate originating from exercise. Aerobic endurance exercise in animal studies shows that there might be a threshold of exercise-induced oxytocin which may strengthen these cardioprotective effects (Hada et al., 2003; Lesimple et al., 2020; Tolentino Bento da Silva et al., 2021).

Cross-reading on the potential yields of exercise from animal studies is difficult, humans may perceive even an ultramarathon as voluntary and prolonged exercise. Interestingly, during prolonged exercise, the correlation of the antidiuretic hormone-arginine vasopressin (AVP) and oxytocin plays a role in social recognition which is crucial for human and animal relationships and mating (Hew-Butler et al., 2008a; Hew-Butler et al., 2008b; Zeng et al., 2012). Subsequently, being healthy carries mating benefits in humans and animals but this relationship warrants further studies (Zeng et al., 2012; Kenkel and Carter, 2016).

Another study found that martial arts may increase oxytocin. The research particularly examined different forms of martial arts, like ground grappling and “punch-kick” sparring. Insight into how intense physical and interactive exercises like martial arts impact oxytocin levels is crucial, as touch is a complicated phenomenon in sports science since the type, frequency, and valence of touch can alter oxytocin responses (Rassovsky et al., 2019). Although “Dennis Jiu Jitsu” as such, is a non-traditional and questionable martial art activity because of its belt system and utilization of various martial arts, the study’s findings on oxytocin are of high value.

Sport types, however, can differ widely. As an example, dance and rhythm might be able to affect humans through the mirror neuron system and physical touch, which has an established connection with empathy and oxytocin (Heinskou and Liebst, 2016). Unfortunately, interaction with auditory stimuli might make it challenging to explore sports connected to music (Jezova et al., 2013). Oxytocin plays a role in how athletes perceive and respond to competitive stress, linking psychological states with physiological responses (Meijen et al., 2020). Furthermore, higher oxytocin levels, influenced by perceived social support, may lead to a “challenge state”, enhancing athletes’ self-esteem and improving performance.

Team sports therefore open many possibilities for investigation, as a single dose of intranasal oxytocin can change how people perceive each other, even from their body language (Bernaerts et al., 2016). Traditional hunting can be considered as aerobic exercise in humans, which also has shown a significant influence on oxytocin (Jaeggi et al., 2015).

Rugby, which involves high physical contact, and handball, which besides slightly different contact valence requires specialized team coordination, have already been investigated in connection with competitions (Kociuba et al., 2023), however, the half-life of oxytocin was not considered properly when measuring urinary samples days away from the competition itself. A study also recorded that acute hypoxia stimulates oxytocin release from the rat hypothalamus whereas thyrotropin-releasing hormone has an inhibitory action during a stress response. Acute hypoxia can be caused by anemia, asthma, or swimming exercise and training in SD rats (Xu et al., 2006).

In sports, this can occur in activities at high altitudes, such as mountain climbing, skiing, or high-altitude training for endurance athletes, but also in endurance sports like marathon running or cycling, where intense exertion may lead to temporary oxygen deficiency in muscles.

A study of well-established and defined sports on oxytocin levels could yield valuable insights into the relationship between oxytocin and exercise. Consequently, the type of sport may be a crucial factor in determining whether exercise will be sufficient to elicit change in oxytocin. Measurements therefore in sports science, concerning oxytocin, should account for the time, intensity, and environment of exercise so that the threshold of exercise can be documented, and the benefits of oxytocin can be further explored and utilized.

## 2 Methods

PICO key term and research question translated to SPIDER.

Population (P) = Sample (S): Inclusion: healthy adult male population (both animal and human). Exclusion: subjects under the age of 18 or with pre-existing medical conditions, females
Intervention/exposure (I) = The phenomenon of Interest (I): Oxytocin research and methodology particularly focusing on exercise
Comparison (C) = Design (D): Is oxytocin measured? If yes, did it increase or decrease? Did exercise performance increase or decrease due to said release?
Outcomes (O) = Evaluation (E): Involves information on oxytocin that may be connected to exercise. What is their causal relationship?
Research type (R) = English studies published in the last 20 years, randomized controlled trials (RCTs), non-randomized studies: randomized controlled trials, interrupted time series, systematic reviews, controlled before and after studies, and cohort studies were included in the study.

### 2.1 Eligibility criteria

#### 2.1.1 Inclusion criteria

Randomized controlled trials (RCTs), non-randomized studies: interrupted time series, systematic reviews, controlled before and after studies, and cohort studies were included in our investigation. We confined our scope to the English language and studies published over the last 20 years, thus remaining contemporary. Studies were required to have information on oxytocin, measurements, and exercise. Included articles must be directly related to oxytocin, its measurement, sport theory, sports practice, and sport physiology and should provide insights applicable to these areas, while focusing on healthy populations. Through such an explicit approach we will filter out the works that are irrelevant to our research purposes and focus on the ones covering physiological, theoretical, and practical issues in exercise science both in humans and animals.

#### 2.1.2 Exclusion criteria

We have set the exclusion criteria so that the review focuses on the most relevant, reliable, and the most up to date research. Conference abstracts and study protocols usually lack the rigorous peer review process needed for reliability. We did not include study reports that did not fulfill the CONSORT requirements, i.e., conference abstracts. Studies without published results (e.g., published protocols) were excluded too. Also, studies published in languages other than English were excluded since their results could be misinterpreted due to the potential linguistic barriers. A time cut-off was set for the studies published before 2003 to accommodate the review in drawing on recent methodological developments and understanding in the field. The reliability of the measurement strategies utilized in the studies was a crucial factor; hence, to preserve the validity of the review outcomes, any studies that applied unreliable measurement methods were taken out. Also, reviews concentrating on clinically diagnosed diseases were taken out of the review, as the aim was to study the association between oxytocin levels and exercise in otherwise healthy populations. Considering the scope of this review, we excluded studies that were carried out having female subjects only, with a focus on reproduction or on aspects of sexual function. The choice was made for this framework to achieve applicability to a wider demographic subset recognizing their needs separate a detailed investigation due to the complexities of these areas and the effect of states or conditions like menstrual cycle, pregnancy, birth control, and sex on oxytocin levels. In the end, studies that were not immediately applicable to exercise or did not have a direct impact on it were excluded, and the aim was to specifically identify the relationship between physical activity and oxytocin levels, the results thus obtained may be directly applied in the human performance and wellbeing improvement. This targeted approach to selecting studies aims to investigate the relationship between oxytocin and exercise, providing a comprehensive and accurate basis for future studies and applications in this field. The exclusion criteria were attentively made to warrant that the resulting framework is based on the most trustworthy, applicable, and contemporary scientific data.

Studies grouped for the syntheses (outlines the chapters of the review):

1. Exercise (Cognitive Functions and Gene Polymorphism)
2. Physiology (Pain, Analgesia, Injury, and Nutrition)
3. Environment (visual, auditory, and olfactory stimuli)
4. Social Context (physical contact, psychological interventions, and play)
5. Stress (acute, chronic, and restraint)
6. Measurements.

### 2.2 Information sources

These databases were last accessed on 2023-08-01. We searched Scopus, PubMed, Embase, Web of Science, and Ovid Medline. When applicable, MeSH was applied to boost accuracy. All databases were simultaneously accessed, with the results stored in Excel spreadsheets, for both reviewers to work alongside each other, and discuss the downloaded literature and documentation, to take readily available notes. PRISMA guidelines were followed and consulted throughout the study. To systematically generate our questions and keywords, we employed the “SPIDER” methodology. The query strings for Boolean operators were built on each side based on Pubmed’s Query Builder and MeSH database integrated into the advanced search.

### 2.3 Search strategy

Filters applied: (2003–2023) English Only-last 20 years. MeSH was embedded into the keywords to increase search precision (DeMars and Perruso, 2022).

Scopus (68) (No MeSH available): TITLE-ABS-KEY-AUTH ((((“oxytocin”) AND (((((“exercise”) OR (“sport*”)) OR (“physical activity”)) OR (“train*”)) OR (“workout*”))) AND (((“measur*”) OR (“assess*”)) OR (“evaluat*”))) AND NOT ((((((((((((((((((“female”) OR (“mother”)) OR (“maternal*”)) OR (“wom?n”)) OR (“birth”)) OR (“labor”)) OR (“deliver*”)) OR (“childbirth”)) OR (“cancer”)) OR (“disease”)) OR (“disorder”)) OR (“defect”)) OR (“malad*”)) OR (“ill”)) OR (“sick*”)) OR (“infect*")) OR (“syndrom*”)))).

PubMed (68) (MeSH applied): (((“oxytocin”) AND (((((“exercise”) OR (“sport*”)) OR (“physical activity”)) OR (“train*”)) OR (“workout*”))) AND (((“measur*”) OR (“assess*”)) OR (“evaluat*”))) NOT ((((((((((((((((((“female”) OR (“mother”)) OR (“maternal*”)) OR (“wom?n”)) OR (“birth”)) OR (“labor”)) OR (“deliver*”)) OR (“childbirth”)) OR (“cancer”)) OR (“disease”)) OR (“disorder”)) OR (“defect”)) OR (“malad*”)) OR (“ill”)) OR (“sick*”)) OR (“infect*”)) OR (“syndrom*”))).

Embase (57) (MeSH applied): (“oxytocin”/exp OR “oxytocin”) AND (“exercise”/exp OR “exercise” OR “sport*” OR “physical activity”/exp OR “physical activity” OR “train*” OR “workout*”) AND (“measur*” OR “assess*” OR “evaluat*”) NOT (“female”/exp OR “female” OR “mother”/exp OR “mother” OR “maternal*” OR “wom?n” OR “birth”/exp OR “birth” OR “labor”/exp OR “labor” OR “deliver*” OR “childbirth”/exp OR “childbirth” OR “cancer”/exp OR “cancer” OR “disease”/exp OR “disease” OR “disorder”/exp OR “disorder” OR “defect” OR “malad*” OR “ill” OR “sick*” OR “infect*” OR “syndrom*”).

Web of Science (258) (No MeSH available): (((“oxytocin”) AND (((((“exercise”) OR (“sport*”)) OR (“physical activity”)) OR (“train*”)) OR (“workout*”))) AND (((“measur*”) OR (“assess*”)) OR (“evaluat*”))) NOT ((((((((((((((((((“female”) OR (“mother”)) OR (“maternal*”)) OR (“wom?n”)) OR (“birth”)) OR (“labor”)) OR (“deliver*”)) OR (“childbirth”)) OR (“cancer”)) OR (“disease”)) OR (“disorder”)) OR (“defect”)) OR (“malad*”)) OR (“ill”)) OR (“sick*”)) OR (“infect*”)) OR (“syndrom*”))).

Ovid (239) (MeSH applied): ((“oxytocin” and (“exercise” or “sport*” or “physical activity” or “train*" or “workout*”) and (“measur*” or “assess*” or “evaluat*”)) not (“female” or “mother” or “maternal*” or “wom?n” or “birth” or “labor” or “deliver*” or “childbirth” or “cancer” or “disease” or “disorder” or “defect” or “malad*” or “ill” or “sick*” or “infect*” or “syndrom*”)).mp. [mp=ab, bo, bt, ti, hw, tx, mc, st, or, tn, ps, ds, cb, rn, sq, mq, ge, tm, mi, ct, sh, ot, nm, fx, kf, ox, px, rx, an, ui, on, sy, ux, mx].

### 2.4 Selection process

After screening the titles and abstract for applicability, studies had to contain information about oxytocin, and/or exercise-related information that was directly relatable and applicable. Color-coding and categorizing the papers was independently done by two reviewers, and subsequent discussions were before our chosen mediator. We found 455 pieces of literature in total, out of which 39 were debated for inclusion. We ended up with 175 papers as full-text reviews. In addition, all decisions were documented with data validation to avoid manual mistakes and to enhance clarity.

### 2.5 Data collection process

Microsoft Power BI (Becker and Gould, 2019) was used to gather all additional and missing abstracts, and information based on DOI numbers and PubMed IDs, to have a comprehensive dataset in Excel, through the utilization of the site’s respective APIs and in the case of open-access manual web scraping. The coding and the process are documented and can be made available if necessary. Two reviewers worked simultaneously, if possible, sometimes independently, with tracked changes and notes on a cloud-based Microsoft Excel dataset. The Power BI procedure, which was strengthened by a script-skipping mechanism, ensured that accurate data was retrieved on the abstracts. However, we recognized the inherent constraints of automated data collecting and performed manual checks to ensure thorough analysis.

Through structural and cross-thematic analysis we investigated 175 pieces of literature after applying our inclusion-exclusion criteria. Oxytocin measurement methodologies and psychological tests were screened in each text. Time points, analyses, and measurements were meticulously recorded in the “Measurement” section, compiling a comprehensive dataset, based on which we collected and evaluated the findings.

Our aim was to be as precise as possible in building a framework for measuring oxytocin and understanding the relation with exercise. The relevance of the results and potential applicability to females, necessitate additional investigation, and critical evaluation. While animal studies predominated due to the existing research gap in oxytocin, this fact was duly noted.

### 2.6 Study risk of bias assessment

The risk of bias assessment was crucial for us in determining the internal validity of the included studies; this is of paramount importance in our analysis. We evaluated numerous aspects of the research papers, such as the sample, protocols, and study types to determine whether the investigated articles had any methodological problems or biases. Generally, to “lower” the risk of bias means that the evidence is more trustworthy. The risk of bias was evaluated using standardized tools. RoB 2 (The Risk of Bias 2) for randomized controlled trials, ROBINS-I (Risk of Bias in Non-randomized Studies—of Interventions) for comparative studies, and ROBINS-E (Risk of Bias in Non-randomized Studies—of Exposures) for cross-sectional and cohort studies. Depending on the type of observations, case-control studies were assessed with either ROBINS-E or ROBINS-I. Assessment of reviews was performed using ROBIS (Risk of Bias in Systematic Reviews assessment tool) tool, and all the animal studies were assessed according to SYRCLE’s (Systematic Review Centre for Laboratory Animal Experimentation) tool. Two reviewers performed the risk of bias assessment jointly, and in case of disagreement, a third reviewer was consulted. Where available, predefined automatization tools were used (through Microsoft Access for ROBIS, and Microsoft Excel for RoB 2 and ROBINS-E). ROBINS-I and SYRCLE were also prepared in Microsoft Excel to enable proper analysis of results (Higgins et al., 2024; Sterne et al., 2019; Hooijmans et al., 2014; Whiting et al., 2016; Sterne et al., 2016).

We performed a narrative, cross-thematic, and structural analysis of the included research papers (Campbell et al., 2020). The analysis of these factors is detailed in the discussion chapter. We conducted the narrative synthesis by searching for oxytocin in each study for the initial coding while noting what parts of the text might contain appropriate data and copying it to the shared Excel dataset. Later we automated the coding process using Power BI for a reliable frequency analysis with data validation via Power Query (Becker and Gould, 2019).

Due to the heterogeneity of the included studies, multiple tools were needed to perform an adequate risk of bias assessments. For the synthesis of the risk of bias assessment, studies were grouped based on the type of the study to produce comparable results. However, outcomes and results of the included studies were presented in a narrative form as this was decided as the most suitable and comprehensible way for synthesis.

**FIGURE 1:**
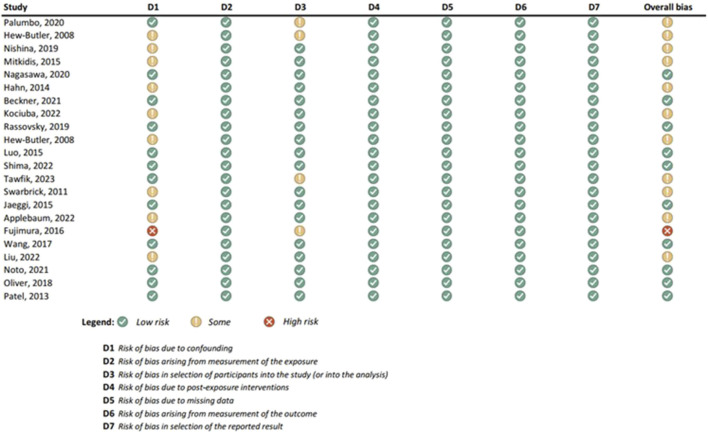
Results of individual study assessments done with ROBINS-E.

**FIGURE 2:**
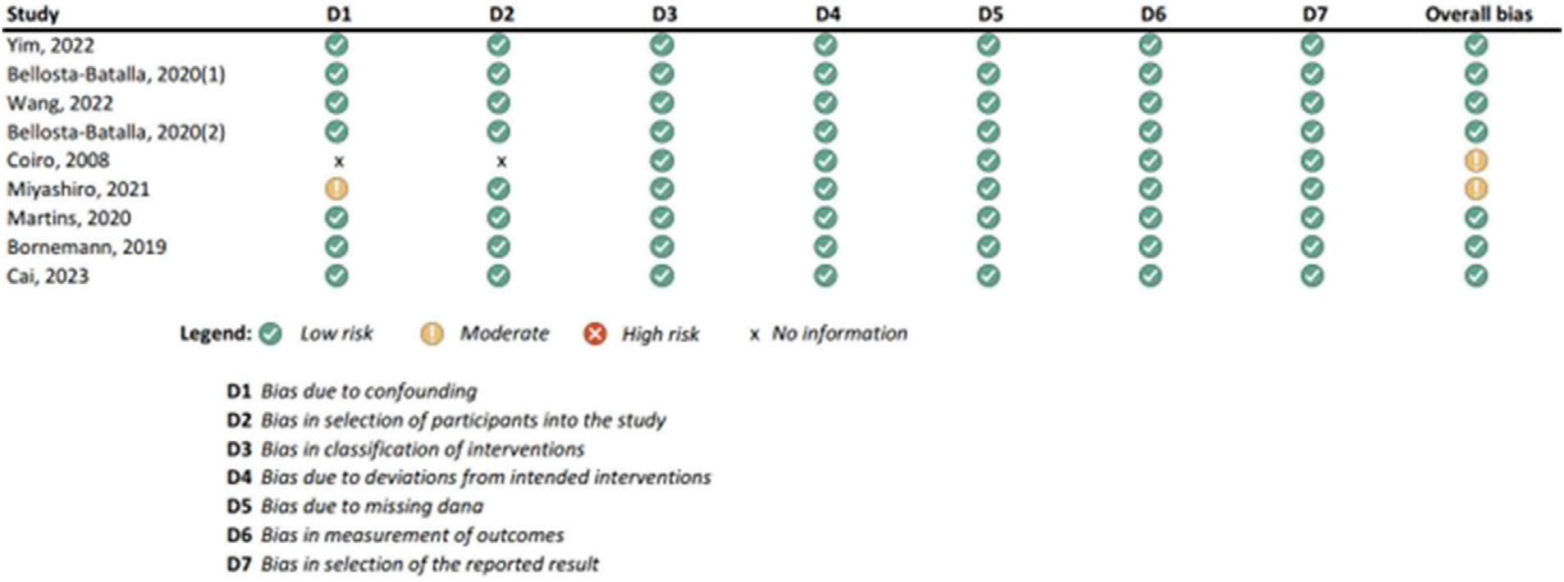
Results of individual study assessments done with ROBINS-I.

**FIGURE 3:**
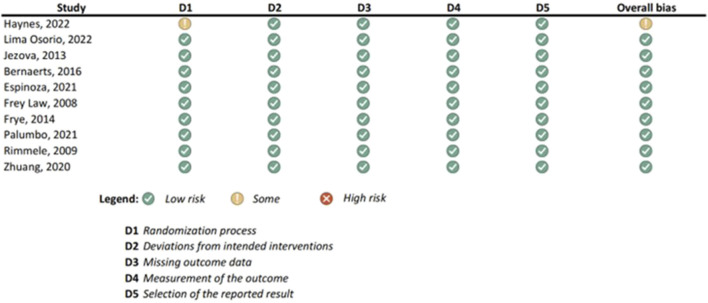
Results of individual study assessments done with RoB 2.

**FIGURE 4:**
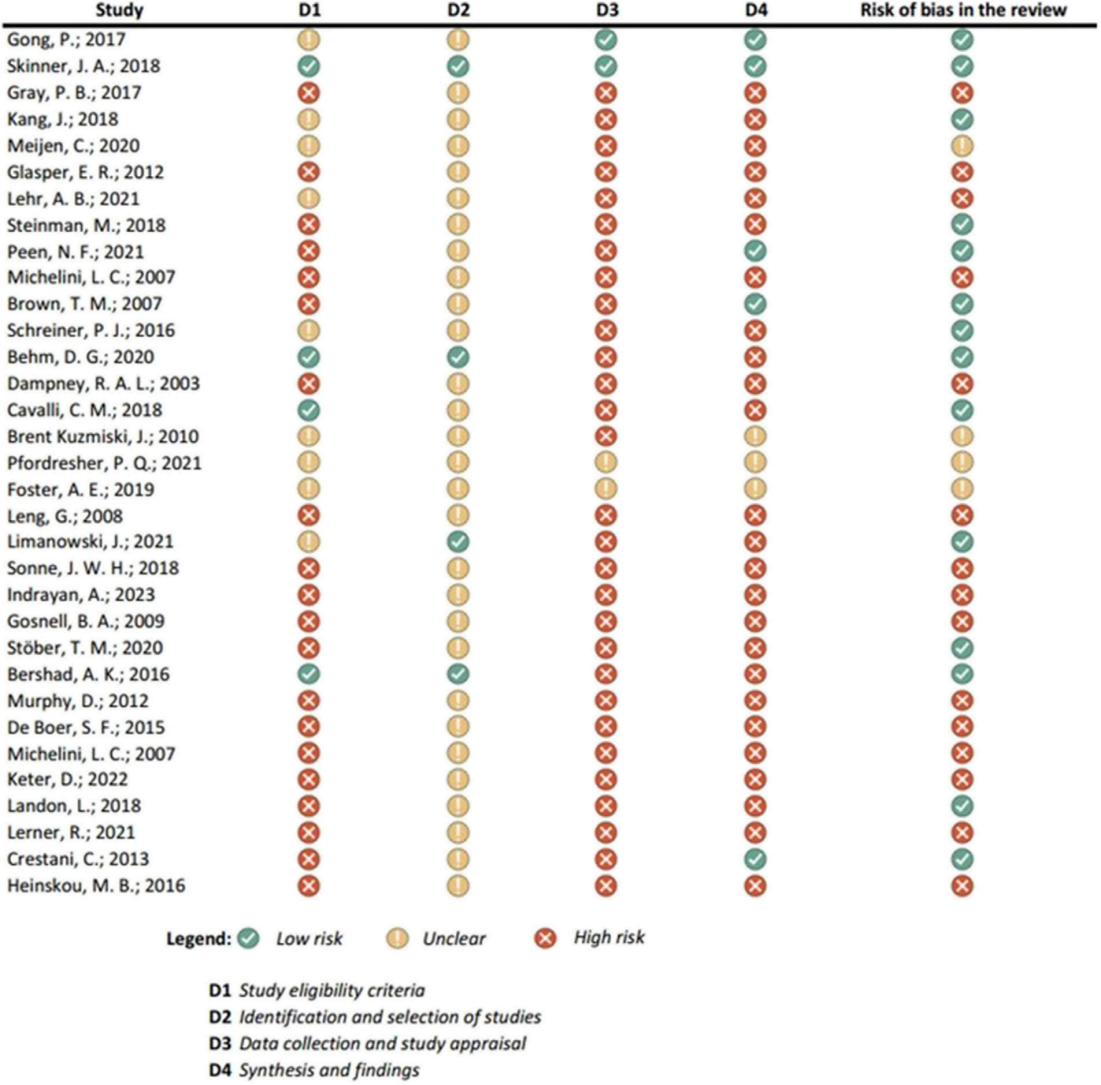
Results of individual study assessments done with ROBIS.

**FIGURE 5:**
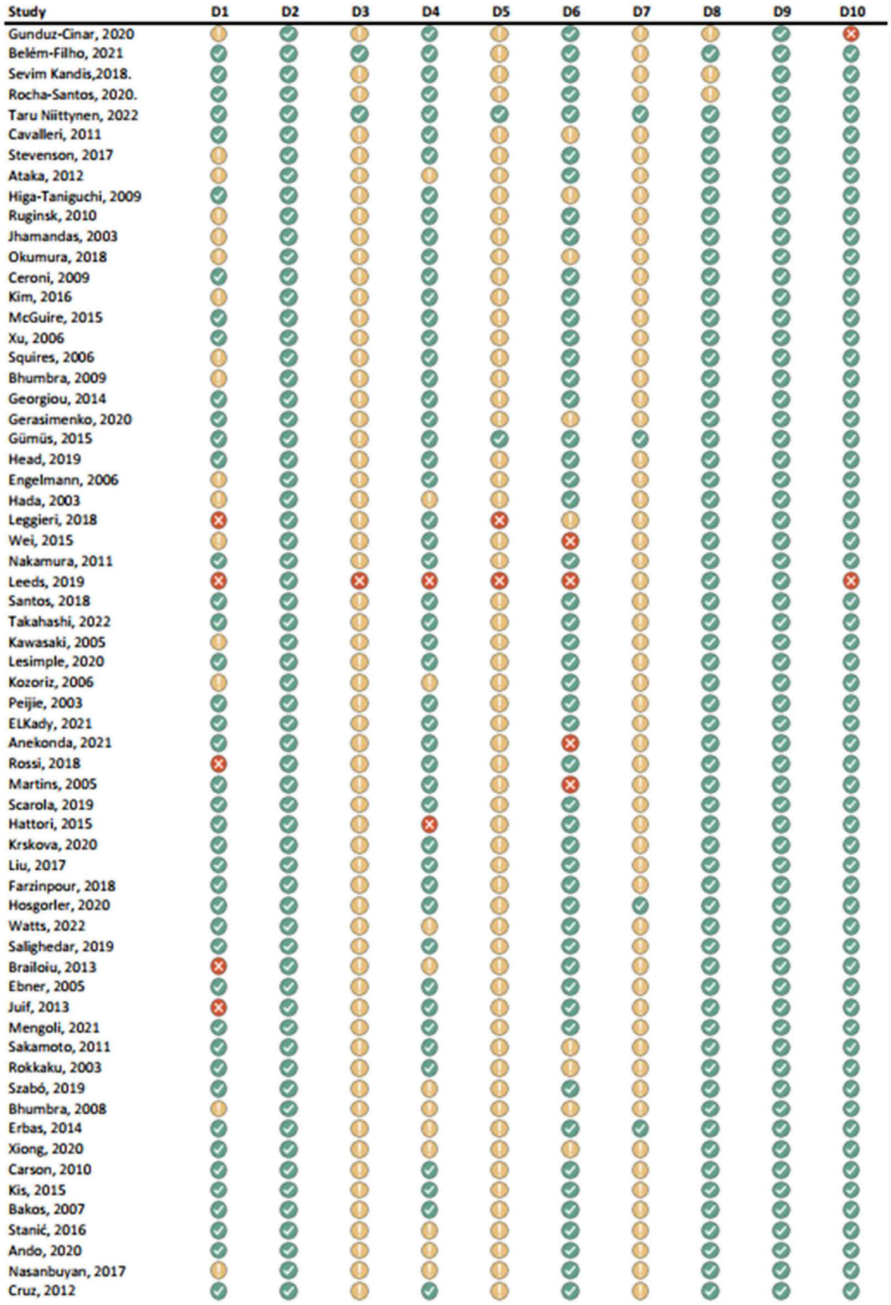
Results of individual study assessments done with SYRCLE (1).

**FIGURE 6:**
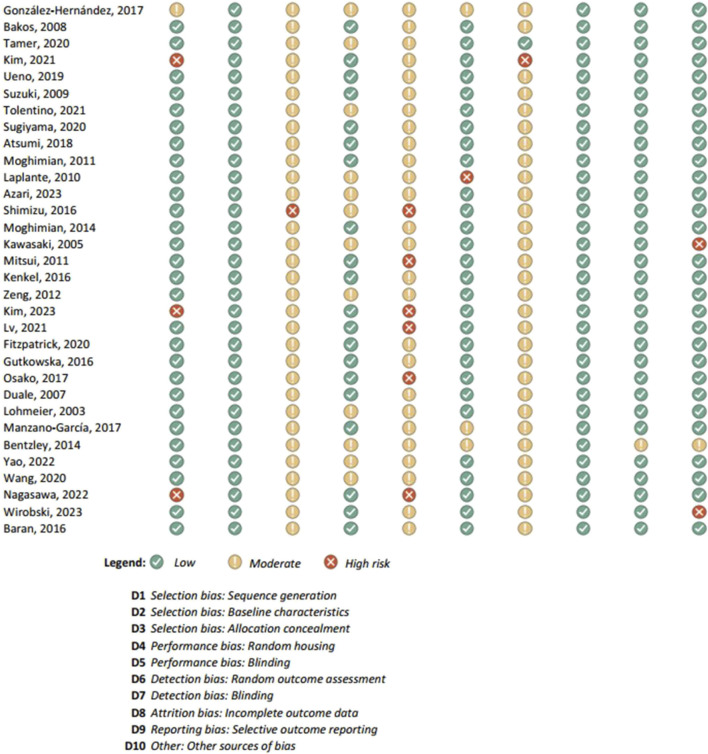
Results of individual study assessments done with SYRCLE (2).

Results of the risk of bias assessments were visually presented using Microsoft Excel, and grouped by tools (study types), to ensure comparability of the results (see Figures 1-6). No meta-analysis has been conducted. Power BI was adopted to further analyze the mass of the screened studies. Moreover, descriptive statistics were used due to having number of years and frequency of each type of study. Average year of publication is around 2015.73 there being an average of studies being published in the middle of 2010. The median year of publication: 2017, which implies that there are as many studies published within the 2017 and after 2017 years and thus, put the median year of publication at the end of the 2010s. Summarizing these statistics, most of the works on oxytocin in the sporting sphere are relatively current with a peak of studies in late 2010-ies. This reveals that as the studies continue to evolve, in general, the last two decades show that understanding of the relationship between oxytocin and exercise has deepened. With regards to oxytocin outcomes, (53) 55.79% of studies indicated that oxytocin increased due to exercise, while (7) 7.37% of studies indicated a decrease in oxytocin (mostly due to chronic physiological conditions); (13) 13.68% of studies indicated that oxytocin improved exercise performance, while (1) 1.05% indicated that oxytocin decreased exercise performance (exercise was assigned as novel a stressor); (21) 22.11% of studies indicated that oxytocin was administered either intranasally or with injection. (see interactive Supplementary Material for cross-filtering).

As for study types: (93) 53.14% of the studies were SYRCLE, (36) 20.57% were ROBIS, (20) 11.43% were ROBINS-E, (11) 6.29% ROB2, (9)5.14% ROBINS-I, (6) 3.43% In-Vitro. Regarding exercise types 63 studies contained no contact exercise, 9 contained contact; 60 were aerobic exercise, 9 anaerobic; 8 contained explicit exploratory behavior; 4 restraint stress or restricted movement; 3 exercise programs. To determine the quality of the included studies, a risk of bias assessment was performed for each study with a tool suitable for the study type. Considering the variety of studies included in the review, RoB 2, ROBINS-I, and ROBINS-E were used for evaluating human studies; SYRCLE’s tool for animal studies and ROBIS tool included reviews and meta-analyses. To present the results of the risk of bias assessment, studies were divided into subgroups (one for each tool). *In vitro* studies were from 2009, 2018, 2020, 2022, and 2023. ROB2 studies were from 2008, with a notable presence in more recent years such as 2020, 2021, 2022, and 2023. ROBINS-E ranges from 2008 to 2023, with a peak in recent years, indicating continued relevance. ROBINS-I appeared in specific years such as 2008, 2019, 2020, 2021, 2022, and 2023. ROBIS has a broad range from 2003 through 2023, consistently, particularly in more recent years. SYRCLE extensively showed up from the earliest years in the dataset (2003) up to 2023, with a peak in usage in 2020.

Attrition bias was appraised by assessing the completeness of data reporting and the handling of missing data. Studies with a low risk of attrition bias provided clear explanations for participant withdrawals and employed appropriate statistical methods to address missing data. The risk of reporting bias was considered low for studies with pre-specified outcomes and adherence to reporting guidelines. Summary statistical information and assessments of certainty are presented in a GRADE evidence manner (Zhang et al., 2019; Guyatt et al., 2011). To ensure the quality of included studies, a risk of bias assessment was performed using the tools mentioned above. All tools aimed to assess several aspects of the study. All studies were scrutinized for any indications of selection bias, such as inadequate randomization procedures or insufficient allocation concealment.

The risk of performance bias was assessed by examining the blinding procedures implemented in each study. To evaluate the potential for detection bias, the clarity and adequacy of outcome assessments were carefully examined. Clearly defined domains examined by each tool are listed in the tables with the results of the risk of bias assessments. Most of the studies were assessed as low risk of bias, still, it was not possible to make a clear judgment in the case when the study authors did not provide enough details, and these studies were marked as unclear. Although part of the reviews and animal studies were rated as high risk of bias, it was decided to include these studies in the review, to gather all potential data for careful evaluation. Several reasons influenced this decision. Sometimes due to the nature of their design, in the case of observation of wild animals and free groups, animal studies could not have clearly defined control groups. Such studies exhibited a higher risk of bias, but we still believe that they provide important insights into the dynamics of oxytocin and the factors that influence it, therefore their results are also included in this review, with the emphasis that they cannot be as reliable as the results obtained by RCTs (randomized-controlled trials) carried out on laboratory animals. Reviews assessed as high risk of bias, sometimes due to earlier publication, were insufficient in terms of defining the protocol, but they provided basic knowledge on this topic. Thus, we deemed it important to include them and consider their contribution to our overall understanding of oxytocin. A detailed risk of bias assessment for each included study, as well as an overview of the risk of bias for each of the study groups, are presented graphically.

## 3 Results

### 3.1 Search and selection process findings

**FIGURE 7:**
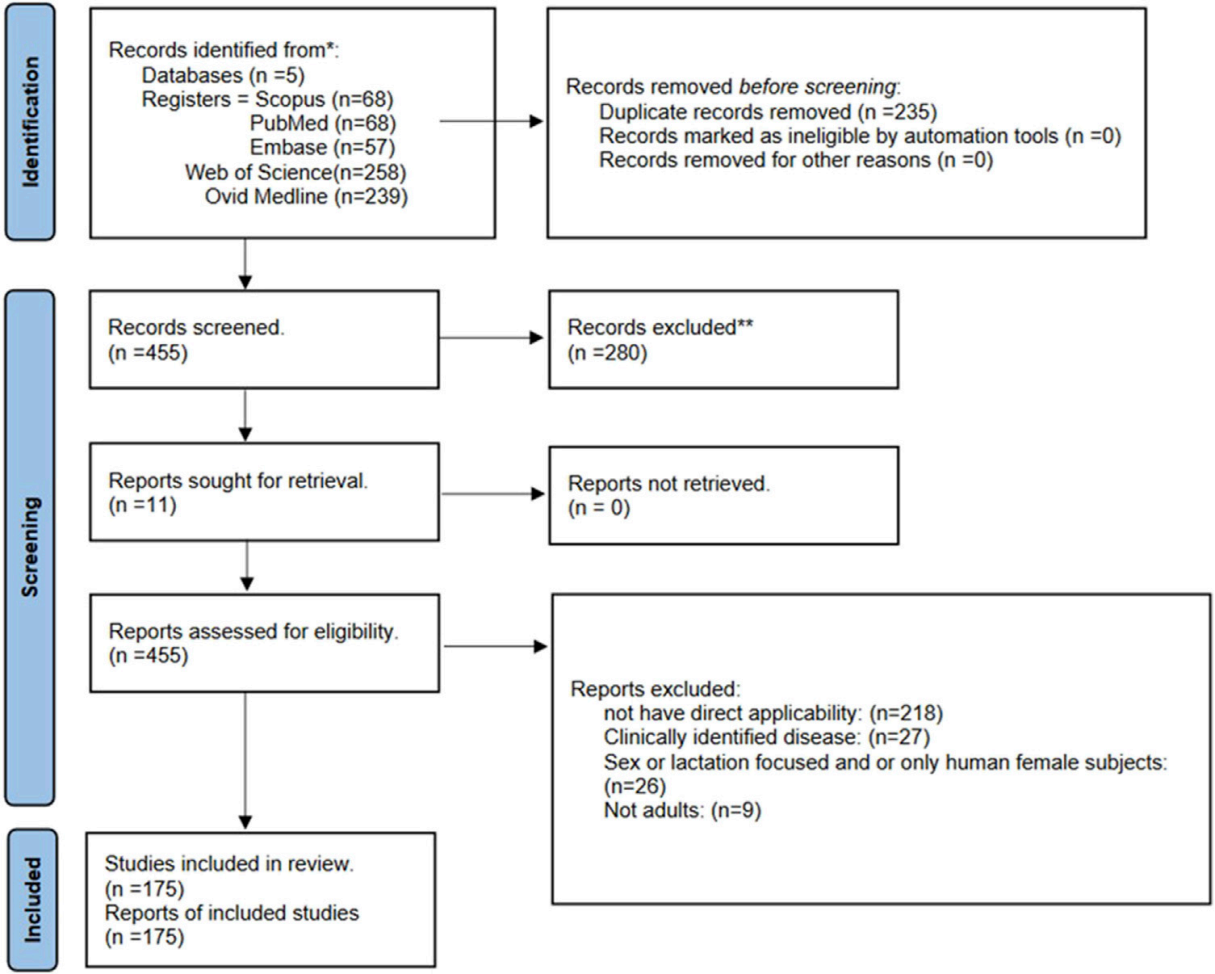
*Flow diagram* (based on Page et al., 2021).

175 studies met our inclusion criteria (see Figure 7). During our structural analysis of the study’s “*Methods*” sections, we observed the following:

#### 3.1.1 Psychological measurements

In our structural analysis, we found 63 psychological and or behavioral tests. After the assessment, 4 self-report tests accounted for the chapters we discussed in our review, potentially enhancing data filtering precision. Given that these tests are taken, not only does the data filtering become more reliable, but the emerging correlations may yield important data for researchers.

1. Perceived Stress Scale (PSS) (Nielsen et al., 2016) to account for stress (aiding in understanding the potential impact of stress on oxytocin levels, or how oxytocin may influence stress levels following an exercise intervention).
2. Multidimensional Scale of Perceived Social Context (MSPSS) (Zimet et al., 1988) for social context, shedding light on potential associations between oxytocin levels and social environments or their absence.
3. Numerical Rating Scale (NRS) (Karcioglu et al., 2018) for pain, investigating the impact of pain experienced by athletes before, during, and after exercise on oxytocin levels and their fluctuations.
4. Council of Nutrition Appetite Questionnaire (CNAQ) (Hanisah et al., 2012) or Food Frequency Questionnaire (FFQ) (Shahar et al., 2003) as an alternative, for nutrition-related data.

#### 3.1.2 Physiological measurements

In the following, we documented the layout of studies that dealt with physiological measurements and observed their occurrence from the total number of included studies (175) alongside oxytocin’s sport related outcomes, so we could observe how measuring methods have been applied throughout the scientific realm.

Wefound thirty-three (32.67%) studies analyzing physiological measurement techniques likeanimal testing with surgery (immunochemistry or fluorescence).

Five (4.95%) studies examined urinary oxytocin, and two (0.99%–0.99%) studies examined utilized fMRI and oxytocin from hair samples respectively.

As for interventions, thirteen (12.87%) of studies utilized oxytocin injections, and six (5.94%) of studies introduced oxytocin intranasally.

Corresponding with the literature we conclude that plasma oxytocin is the most reliable sample when analyzed with radioimmunoassay (Oliver et al., 2018), which is also backed up by the frequency of its occurrence as we found thirty-two (31.68%) of them in the number of analyzed studies.

We found salivary oxytocin in ten (9.9%) studies as a replacement might solve the problems of plasma oxytocin measurements. Admittedly, needles may induce fear and anxiety (Olatunji et al., 2010) which may affect oxytocin in athletes. In addition, salivary testing is also non-invasive and has been done in a sports context (Rassovsky et al., 2019). At the same time, salivary oxytocin analyzed with ELISA kits if internally validated, sampled, and analyzed repeatedly has been a basis for reliable and trustworthy studies as a source of information published in reputable journals (Hew-Butler et al., 2008b; Coiro et al., 2008; Keenan Gerred and Kapoor, 2023; Leng and Ludwig, 2008; Mabrouk and Kennedy, 2012). Keeping up with the test manufacturer’s instructions remains a crucial part of measurements and testing and may prevent possible inaccuracies in measurement procedures (Hew-Butler et al., 2008a; Asano et al., 2018; Gan et al., 2023; Niittynen et al., 2022).

Regarding oxytocin outcomes associated with exercise, fifty-three (55.79%) studies indicated that oxytocin increased due to exercise, while eight (8.42%) studies indicated a decrease in oxytocin (exercise was assigned as a novel stressor); thirteen (13.68%) studies indicated that oxytocin improved exercise performance andtwenty-one (22.11%) studies indicated that oxytocin was administered either intranasally or with injection. (see interactive Supplementary Material for cross-filtering).

### 3.2 Studies that might appear to meet the inclusion criteria, but which were excluded, and explanation of their exclusion

Massage therapy in its essence is widely considered one of the most effective methods for post-exercise recovery. In contrast, there is limited literature on the effects of spinal manipulation and pose greater risks especially in hands of inexperienced practitioners. Regarding spinal manipulation’s standard practices to this day if not done by a doctor are disputed, and the beliefs of athletes seem to matter a lot considering outcomes. It is more commonly used for more severe injuries or illnesses, rather than otherwise healthy athletes; unlike some other aspects of physiotherapy, this is more limited as it can be done only on specific joints—which makes it challenging to point out the exact effect on athletes. Since spinal manipulation is often a treatment of some disorders, it’s even harder to find relevant literature that will help decide whether to include such papers or not—possibly even how to interpret them (Kovanur-Sampath et al., 2017; Plaza-Manzano et al., 2014).

### 3.3 Study characteristics (see also references)

Characteristics of the included studies may be found in the Supplementary Material, containing doi numbers and details named “Database_OT_Review” Excel database, along with conclusions and reviewer information and inputs.

### 3.4 Risk of bias in studies: present assessments of risk of bias for each included study

Out of a total of 175 selected studies, 10 studies were evaluated with the RoB 2 tool, ROBINS-E was suitable for 22 studies, and ROBINS-I for 9 studies. A total of 33 reviews were analyzed using the ROBIS tool, and the SYRCLE tool was used for 95 animal studies. The remaining 6 *in vitro* studies were not assessed for risk of bias since this type of study is not subject to the standard errors that are tested by the risk of bias assessment tools. With the aim of comparability of the results obtained using individual tools, and to ensure sufficient precision and accuracy, the results of individual assessments are presented in groups where the studies are grouped according to the tool used for assessment, i.e., according to the type of study. All results are presented graphically in the manner of a traffic-light plot, and according to groups, they are combined into line graphs (both made using Microsoft Excel). A detailed risk of bias assessment for each included study, as well as an overview of the risk of bias for each of the study groups, are presented graphically.

### 3.5 Results of syntheses

**FIGURE 8:**
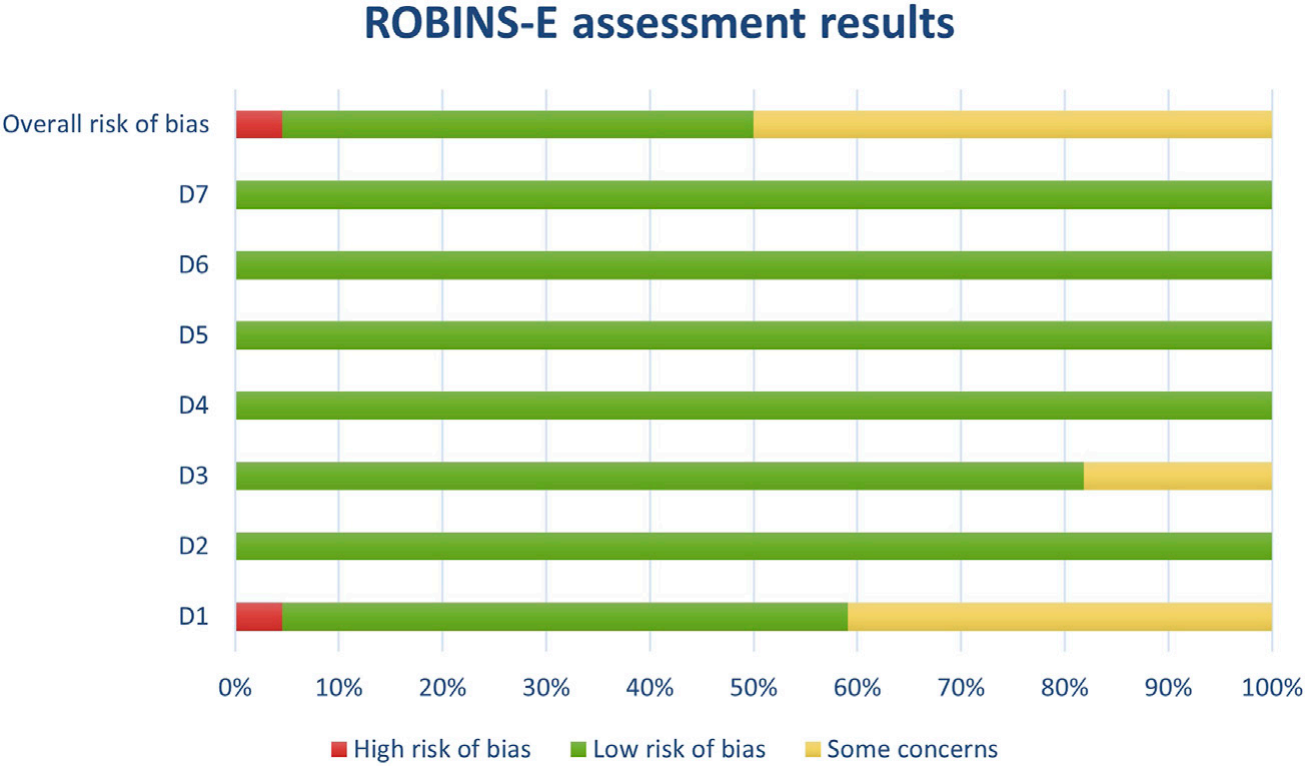
Results of overall risk of bias assessments for studies assessed with ROBINS-E.

**FIGURE 9:**
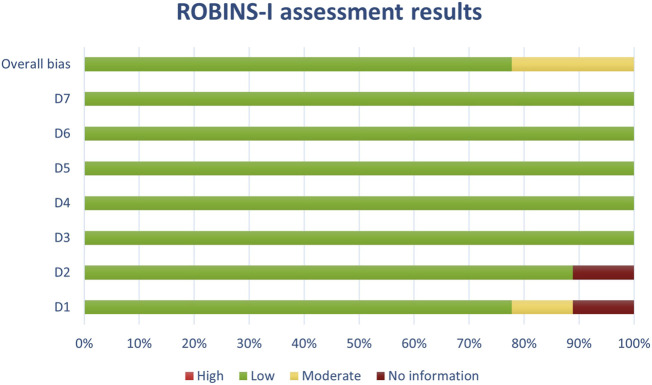
Results of overall risk of bias assessments for studies assessed with ROBINS-I.

**FIGURE 10:**
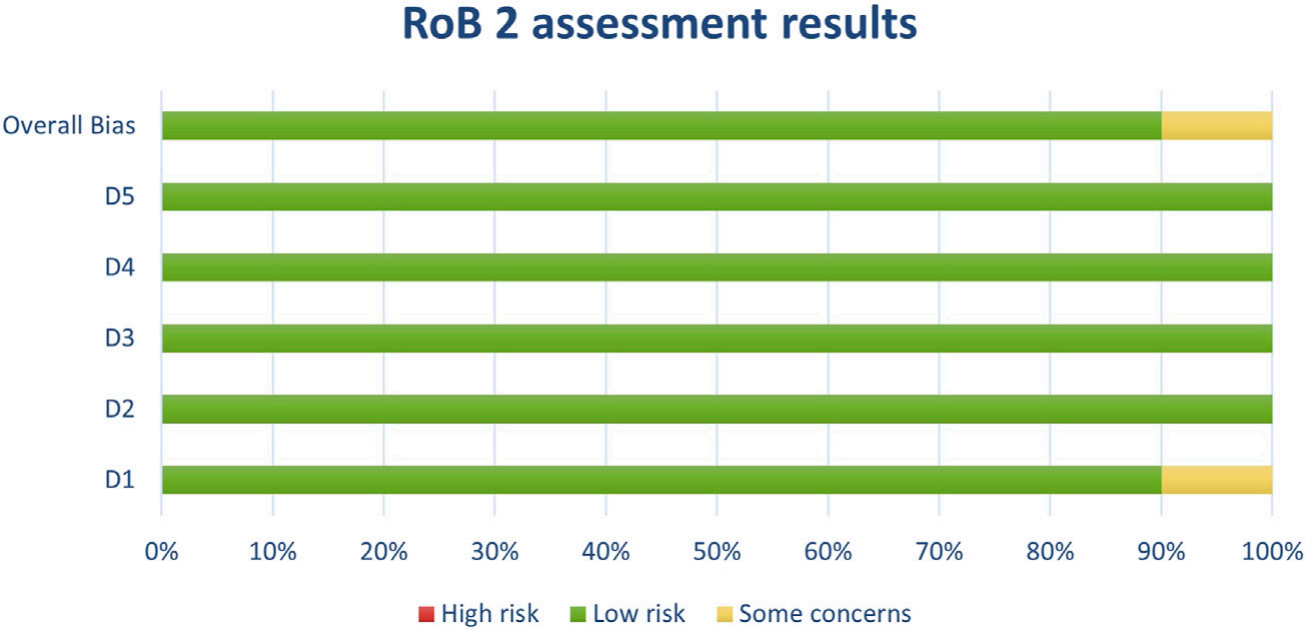
Results of overall risk of bias assessments for studies assessed with RoB 2.

**FIGURE 11:**
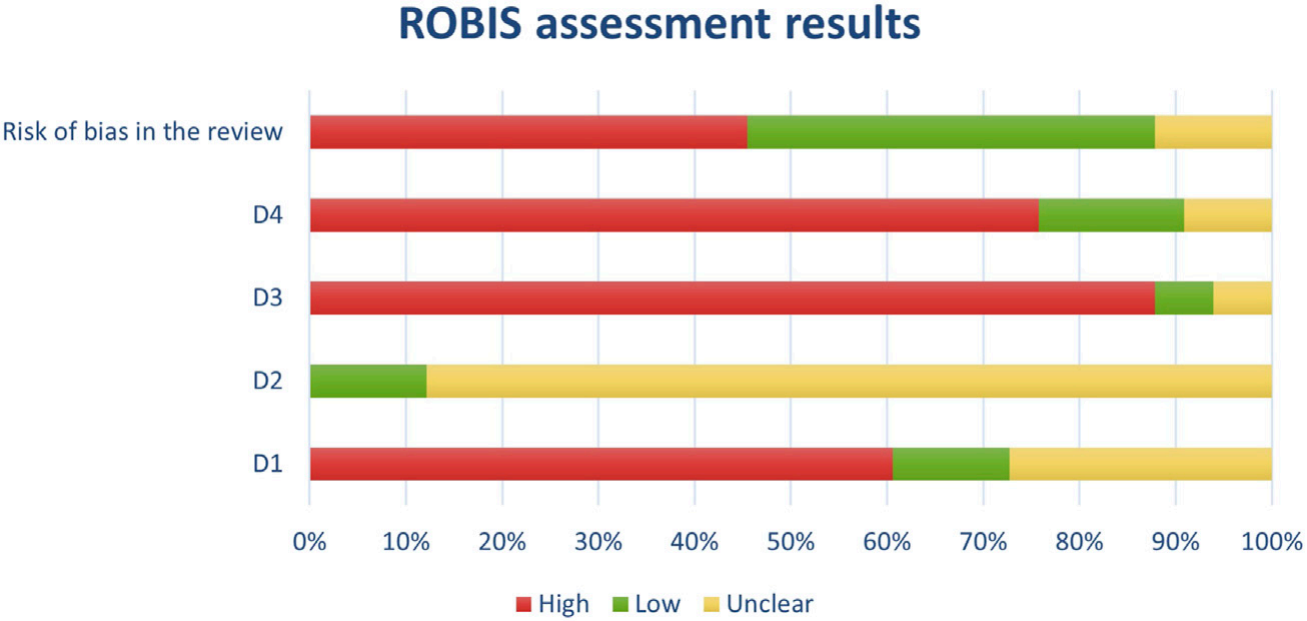
Results of overall risk of bias assessments for studies assessed with ROBIS.

**FIGURE 12:**
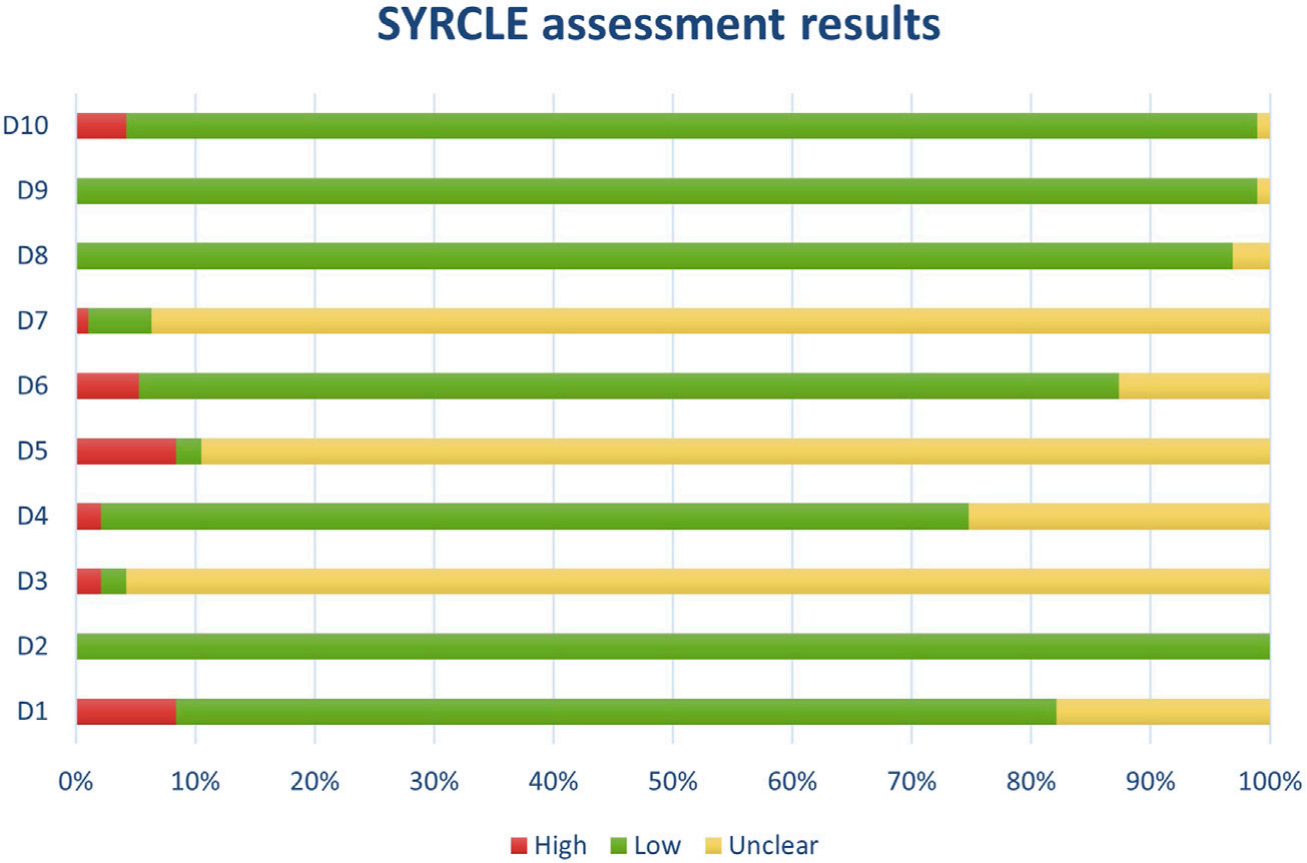
Results of overall risk of bias assessments for studies assessed with SYRCLE.

Synthesis of risk of bias assessments is presented graphically (see Figures 8-12). Animal studies (assessed with SYRCE’s tool) and RCTs (assessed with RoB 2) showed the lowest risk of bias. This is due to the controlled environment in which these outcomes were observed and adequately generated control groups. All the used tools were up to date, and all programs that we used showed no signs of improper functioning. The sensitivity of the involved tools is high all following MECIR (Hoffmann et al., 2014) guidelines.

### 3.6 Reporting biases

Fortunately, we had access to all of the studies during the assessment. In cases of missing data or articles, we contacted our colleagues and co-authors who had access to the appropriate databases to retrieve the full texts of articles and or Supplementary Material for a comprehensive analysis, after double-checking whether the doi and paid identifiers matched with our database we assessed the papers in accordance to the above-discussed protocols.

## 4 Discussion

### 4.1 Pain and analgesia

In our analysis, we reviewed 32 studies that provided information on injury, pain, and inflammation related to oxytocin. Oxytocin also plays a role in rehabilitation, which is supported by animal studies investigating face/mouth and liver damage (Michelini, 2007a; Michelini, 2007b; Moghimian et al., 2012; ELKady et al., 2021; Jhamandas and MacTavish, 2003; Yokoyama et al., 2009; Moghlmian et al., 2014; Okumura et al., 2019; Xiong et al., 2020; Anekonda et al., 2021). Additionally, oxytocin’s recognized anti-inflammatory properties and its ability to facilitate tissue regeneration against oxidative stress have been recognized in rat studies (Wang et al., 2020; Frey Law et al., 2008; González-Hernández et al., 2017; Arabacı Tamer et al., 2020). Furthermore, the degree of inflammation seems to be linked directly to oxytocin in a dose-dependent manner in rats (Juif and Poisbeau, 2013; Bhumbra et al., 2009; Erbas et al., 2014; Indrayan et al., 2023). Exogenous oxytocin may have the ability to lower muscle temperature in mice and rats (Watts et al., 2022), inevitably highlighting the importance of temperature and other variables. Based on this data, it might be possible that oxytocin later could serve as a complementary therapy for sports injuries.

The level of physical contact in sports may influence the social context, subsequently impacting the amount of oxytocin released, indirectly influencing results in both SD rats and humans (Pressman et al., 2013; Luo et al., 2015; Osako et al., 2018; Damian et al., 2022). Therefore, variations in individual differences and social contexts, e.g., loss of partner among humans, and prairie voles may also affect perceived pain and pain thresholds (Kenkel and Carter, 2016; Osako et al., 2018).

Meditation, often practiced before or as part of exercise routines, can likely contribute to increased oxytocin levels. Additionally, oxytocin might be “consumed” for pain reduction resulting in varied individual values (Miyashiro et al., 2021). The presence of meditation and mindfulness is crucial to precision in measurements, as they can potentially alter the perception of pain induced by sports-related oxytocin based on salivary measurements.

Examining correlations between exercise-induced oxytocin and pain could provide insights into the role of oxytocin in nociceptive mechanisms. To address the variability in pain, it is essential to consider individual pain perception, perceived social context, and the level of physical contact. External variables such as obesity in rats (Krskova et al., 2020) and drugs like paracetamol and benzodiazepines, which may be able to mimic oxytocin functions by alleviating anxiety and pain, therefore decreasing oxytocin because of its function being fulfilled, may also impact pain perception in prairie voles and SD rats (Ruginsk et al., 2010; Stevenson et al., 2017; Kandis et al., 2018).

Oxytocin may help athletes in the recovery process, after training or injury by reducing inflammation and promoting tissue regeneration, although this has not been thoroughly tested yet on humans. External factors including meditation and the use or abuse of certain substances, potentially altering oxytocin levels or mimicking its effects by decreased anxiety, inflammation, or pain, sports-related social interactions and physical contact may influence oxytocin release. Subsequently, this influences the psychophysiological state of athletes, which needs to be addressed to make measurements more precise. With self-report tests such as the Numerical Rating Scale (NRS) and the Multidimensional Scale of Perceived Social Context (MSPSS), we were able to consider these variables alongside the possibility of reporting the loss of a partner and commonly used substances (caffeine, tobacco, marijuana, painkillers, etc.) and examine the correlations. Admittedly, this may yield invaluable insights into the relationship of this neuropeptide to pain. In summary, understanding the depth of this relationship could help recovery protocols, training, and conditioning strategies for athletes.

### 4.2 Environment

Changes in environmental conditions such as temperature, olfactory, visual, and auditory stimuli, could influence oxytocin levels. Clarifying the relationships of these variables and then later optimizing for the secretion of oxytocin may be useful data for medicine, sports science, and education. Although there is not much data on visual stimuli, and accommodation periods connected to oxytocin with exercise; studies mention these variables as parts of other protocols (Cavalleri et al., 2011; Farzinpour et al., 2019; Bhumbra et al., 2009; Ataka et al., 2012; Ueno et al., 2019; Liu et al., 2022) warranting further exploration on the subject. The controlled environments in animal studies offer valuable insight into how the behavior of oxytocin may be changed in Long-Evans (LE), SD, W rats, and mice that carry a knock-out allele for the Nms gene. The majority of animal experiments involved in this chapter note all the standard settings in an environment which is also a marker of their reliability (Sakamoto et al., 2011; Cavalleri et al., 2011; Farzinpour et al., 2019; Takahashi et al., 2022; Jhamandas and MacTavish, 2003; Okumura et al., 2019; Arabacı Tamer et al., 2020; Bhumbra et al., 2009; Ataka et al., 2012; Ueno et al., 2019; Dampney and Horiuchi, 2003; Wei et al., 2015; Manzano-García et al., 2018; Head et al., 2019; Salighedar et al., 2019). Consistent cold storage for oxytocin, impacting its effectiveness, and keeping the samples we collect from athletes, will be crucial during the measurement (Oliver et al., 2018).

Exercise intensity can differentially affect oxytocin pathways in the brain, indicating a complex relationship between physical activity and oxytocin levels. In controlled environments, prairie voles (Kenkel and Carter, 2016) and W rats were subject to structured exercise programs, both affecting markers of oxytocin (Tolentino Bento da Silva et al., 2021). These studies show that differences in exercise intensity in both animals and humans may result in distinctive oxytocin responses. Admittedly, this could be invaluable in designing sports programs. The differential effects observed in WKY and SHR rats also point to the need for a punctual analysis of individual health statuses in sports-related oxytocin research (Martins et al., 2005). Furthermore, hard endurance training in SD rats, involving increased swimming durations and intensities, leads to significant rises in stress hormones and neuropeptides, including oxytocin, in the blood and central nervous system regions. Admittedly, changes suggest that physical exercise can modulate the immune system and stress response, however, careful examination of such phenomena is still required in human subjects who are much more complicated and frequently exposed to other sports-related supplementary therapies (Glasper et al., 2012; Behm et al., 2020). Consequently, the connection of exercise intensity’s impact on health and performance remains crucial, attaching aerobic physical activity to potential immune benefits through hormonal pathways via oxytocin (Peijie et al., 2003).

In uncontrolled environments, a study on sled dogs showed a mild increase in oxytocin due to an exercise routine such as mushing (an aerobic endurance exercise) (Leggieri et al., 2019), however, it’s unclear how much the environment influenced the secretion. Releasing horses from their paddock improved welfare, evidently, reduced stereotypic behaviors, and increased oxytocin levels, suggesting enhanced positive emotions. Whereas later, the values of the horse’s markers (emotional and biological) worsened shortly after. Horses experiencing daily free movement in paddocks showed increased oxytocin levels. This increase indicates that free movement positively affects horses’ welfare (Lesimple et al., 2020). Nonetheless, another study found that it is physical exercise that produced changes in oxytocin (Hada et al., 2003). Still, this will require further exploration because even the person interacting with the horses can elicit change in their physiology. On the other hand, environmental enrichment in LE rats may decrease the consequences of chronic stress. Results showed that chronic stress negatively impacted immune response, but environmental enrichment could mitigate these effects. Acute stress combined with enrichment led to healthier immune and stress regulation profiles. These findings suggest that in human athletes, both the type and context of stress, along with environmental factors, might influence immune responses, underlining the complexity of stress-immune interactions in sports contexts. It remains unclear how visual stimuli would affect oxytocin in humans, (Scarola et al., 2019), calling for future studies.

#### 4.2.1 Auditory

During our analysis, we found 12 papers dealing with auditory, and 5 with olfactory stimuli detailing environment. Most of the time animal studies control for auditory input in relation to oxytocin. At the time of writing this review, there is not enough information about the changes due to said input considering this neuropeptide, but it seems that the relationship between oxytocin and auditory input is species-dependent, namely, SD rats, wolves (a strong hierarchical order), cats (somewhat owner-dependent response), and zebra finch (mesotocin-spatial location of birds among each other) behaved differently (Watts et al., 2022; Baran et al., 2016; Fitzpatrick and Morrow, 2020; Hosgorler et al., 2020; Nagasawa et al., 2022; Wirobski et al., 2023). Humans commonly use singing in many rituals, and it is part of every culture throughout the world. Singing together as a social activity may be able to enhance oxytocin and therefore decrease anxiety (Heinskou and Liebst, 2016; Kang et al., 2018; Pfordresher, 2021). The introduction of oxytocin can help singers enhance performance and decrease anxiety (Osório et al., 2022). The social context belonging to the auditory input can lead to an increase in oxytocin through the mirror-neuron system and rhythmic entertainment when experienced by a group, as these systems are connected to our neurophysiology on an evolutionary basis. Two studies deal with a relationship between physical activity, and music as an intervention in humans, suggesting that massage, skin-to-skin contact, “warm touch,” and music can together help recovery and decrease stress (Bellosta-Batalla et al., 2020; Palumbo et al., 2022) Unfamiliar and unpleasant sounds can lead to decreased oxytocin in humans. Exercise and mental challenge together had no effect on state anxiety when pleasant music was also played simultaneously. On the other hand, state anxiety rose when sounds from the same song were played backward. When state anxiety was higher, adrenocorticotropic hormone concentrations in response to mental challenges were lower, and systolic blood pressure measured alongside a handgrip exercise also decreased (Jezova et al., 2013). Personal preferences, cultural backgrounds, and individual differences in auditory processing might influence oxytocin responses, needing further exploration.

#### 4.2.2 Olfactory

Modulating social behaviors and emotional states, oxytocin is present in key brain areas including the hippocampus and amygdala (Lv et al., 2022). While animal studies show oxytocin’s behavior-modulating effects via olfactory mechanisms, such as inducing aggression or group protection in mice through urinary proteins and a way for individual recognition (Hattori et al., 2015), translating these findings to humans remains complicated because human social interactions and olfactory experiences are far more complex due to the somewhat different brain structure, social norms, and sensory processing. After all, smells and scents can both induce disgust or serve as the basis for a personal approach which are two widely different ways of behavior. Still, mice can discern between stress and relief odors, probing that humans might also detect emotional states through smell, potentially influencing social dynamics (Peen et al., 2021) In humans, oxytocin is known to decrease stress indicators like cortisol, relevant in scenarios combining physical effort with emotional experiences (Mitsui et al., 2011), suggesting that in activities involving both elements, oxytocin levels might rise. Measuring oxytocin levels, for instance, through saliva, can be impacted by various factors from the olfactory environment, and admittedly may be under the influence of circadian rhythms or dietary factors. To control this in animal studies, single housing arrangements and thorough cleaning of apparatuses are used to minimize the diffusion of oxytocin via olfactory mechanisms, and measurements usually take place at the same time (McGuire et al., 2015). Consequently, while animal research offers foundational insights, comprehensive human-specific studies are crucial for understanding oxytocin’s role in social dynamics and its interaction with olfactory cues. Exploring how pleasant/unpleasant olfactory cues and the time of measurement affect oxytocin levels and social interactions may provide valuable insights in the future.

Pavlovian Conditioned Approach (PCA) may not have elicited changes in oxytocin levels in SD rats, possibly due to species-specific differences in cognitive and emotional processing, leaving the possibility open for similar research in humans. Exploring oxytocin levels in team sports or among singers may show how auditory stimuli, such as synchronized and rhythmic cheering or team chants, might influence team cohesion and performance. Considering individual sports, measurement of oxytocin may reveal the impact of the olfactory stimuli, as an example the distinct scents of a gym on an athlete’s stress levels and/or performance. Whereas in rhythmic group exercises such as dance or synchronized swimming, monitoring oxytocin could show the intricacies of collective motion and music improving group cohesion and reducing anxiety. Apparently, we might be able to show and underline the role of the mirror neuron system in activities like dance, in which facial expressions and posture vary depending on styles. Sports psychology and trainers, training facilities could potentially benefit from understanding and manipulating auditory and olfactory stimuli around athletes similarly to how the sound, light, and scents are optimized in-store atmospherics (Spence et al., 2014). In the process of measuring oxytocin, careful consideration should be given to exclude potentially unpleasant stimuli unfamiliar to athletes, as this might lead to more precise measurement outcomes. This approach could open new avenues in optimizing athletic performance and team dynamics. Accounting for environmental changes impacting oxytocin levels is crucial. The temperature, time of measurement, and presence of supplemental therapy (olfactory, auditory), including other instruments (object density), might serve as useful additional information. We understand that athletes may not be placed in a controlled setting just for the purpose of oxytocin measures. However, athletes having their own usual exercise setting might be advantageous to the validity of the scores wherever possible, so that they are accommodated in the same settings.

### 4.3 Cognitive functions

We found 14 studies connected to cognitive functions. Learning and memory are crucial depending on the type and purpose of exercise we engage in. Oxytocin has the potential ability to improve learning and adaptability in humans influencing both positive and negative feedback. This might aid athletes to focus better by reducing distraction from errors and increasing awareness and motivation to correct potential mistakes (Zhuang et al., 2021). Still, individual variations may influence how this will play out in athletes. In the medial prefrontal cortex (mPFC) region of rats' and mice’s brains, oxytocin can improve memory and counter memory impairment caused by morphine (Salighedar et al., 2019), demonstrating that oxytocin in the prelimbic cortex of the mPFC improves memory performance. In addition, oxytocin pretreatment prevented impaired memory functions induced by morphine in this brain region which was tested in a MWM. As of yet, the applicability of the theory on exercise-induced oxytocin to humans remains to be determined. Nevertheless, considering the action mechanisms of oxytocin on memory processing may lead to therapeutic approaches (Salighedar et al., 2019). At the same time, however, this highlights the need for athletes’ self-reporting of whether they consumed drugs or not during a measurement which is addressed in the pain and analgesia, inflammation chapter too. We also found a theoretical study that delved into the role of oxytocin in prioritizing memory sequences in the hippocampus, highlighting its importance in social recognition memory and suggesting a complex interplay between different brain regions and neuromodulators (Stöber et al., 2020) The CA2 and CA3 areas of the hippocampus play a role in deciding what memories to focus on and what to execute. Understanding how oxytocin might affect these areas could lead to better performance in exercise with a focus on technical and tactical development, to facilitate training connected to memory in sports. Social cognition is a crucial part of development in team sports and an essential part of life and learning for humans. In people, oxytocin increased trust in strangers but did not affect the willingness to take non-social risks. This suggests that oxytocin may work by reducing fear responses in the brain (Wang et al., 2017), which inevitably plays a role in any type of sports game and prolonged exercise. We found a study hinting at oxytocin’s link to social bonding in pet owners, which might protect cognitive function in humans, though the exact biological pathways remain unclear (Applebaum et al., 2023). Connected to exercise, the involvement of pets and dogs is more and more common in leisure activities, the presence of which could influence oxytocin secretion. Research on dogs and mice showed that oxytocin enhances social cognition, affecting behaviors like gazing and shoaling. We also found a study indicating oxytocin’s role in processing social cues across different species (Atsumi et al., 2018). A more in-depth look at this paper shows how mice distinguish between biological motion and scrambled motion in the presence of oxytocin. Despite humans being more complex, spatial coordination, strategy, and team interactions may be likewise influenced by oxytocin. In rats, spatial learning in a MWM improved social recognition, potentially due to increased oxytocin and AVP levels. The study suggests that these neuropeptides enhance social behavior following certain learning experiences (Zeng et al., 2012). Through oxytocin pathways, spatial learning activities may affect the performance and social cognition of athletes.

The fear extinction process could be connected to a sports injury or situation where a person or animal gradually loses their fear response to something that used to scare them. Previous studies have investigated the notion that oxytocin helped reduce fear responses in mice and humans, suggesting its potential use in treating trauma-related disorders. Intranasal oxytocin and direct brain infusions in animals showed reduced fear, with a focus on a specific mouse strain (S1) known for poor fear extinction (Gunduz-Cinar et al., 2020). Investigating this further would in all probability provide crucial insights into managing sport-related anxiety, which may help with performance. In mice, oxytocin aids in overcoming fear memories. Furthermore, higher oxytocin and AVP levels can lead to prolonged social memory and excessive grooming, which may serve as a potential distraction from fear responses (Squires et al., 2006). This could occur in humans too when they are under pressure before partaking in a competition, although oxytocin’s interplay here is not clear. In mice, these effects depend on brain region and dosage. Unfortunately, for now, it would be far-fetched to assume a direct translation to humans, but accounting for body language signs also could be a possibility in measuring fear-related oxytocin and social cognition. Oxytocin did not significantly alter cognitive biases or preferences in a modified conditioned place preference test in rats. Oxytocin’s anxiolytic effects may not directly influence cognitive bias, therefore further research is needed on this front (McGuire et al., 2015). Quick decision-making under pressure is an essential part of team sports, and oxytocin’s role in shaping cognitive biases and responses to uncertainty could be relevant.

Oxytocin influences how athletes may process both positive and negative feedback. A more in-depth understanding of oxytocin’s effects on memory and cognitive functions of rats and mice translated to humans could potentially aid mental training and recovery in sports, particularly in technical, tactical development, and mental training. The role of oxytocin in fear extinction and its impact on social cognition is crucial. Managing sports-related anxiety and enhancing team dynamics can be associated with oxytocin’s relation to cognitive functions.

### 4.4 Genes and gene polymorphism

In animals, oxytocin, stress, gene expression, and metabolism show a complex network of relations. In horse breeds, genetic variations may reveal the interplay between oxytocin on trainability, where a correlation is found between trainability, oxytocin, and serotonin (Kim et al., 2023). The neurohormonal and genetic profiles in dogs could reveal roles that they play in humans, as oxytocin is part of dogs’ bonding process related to their gazing behavior (Cavalli et al., 2018; Mengoli et al., 2021), or helped dogs react more positively to ambiguous stimuli (Kis et al., 2015). Oxytocin receptor-null mice exhibited abnormal aggression patterns, suggesting a key role of the oxytocin receptor gene (*OXTR*) in modulating social and competitive behaviors (Hattori et al., 2015). Research on C57BL/6J mice demonstrates that social defeat stress activates oxytocin neurons in key brain regions (Gerasimenko et al., 2020), suggesting a genetic predisposition influencing athletes’ responses to stress and defeat, potentially affecting their resilience and recovery. (Nasanbuyan et al., 2018). This could imply that variations in the human *OXTR* might influence athletes' responses to competitive situations and aggression, impacting their performance and team dynamics. On a metabolic note, the P2Y1 receptor’s role in hormone secretion, particularly leptin in adipocytes, offers insights into how exercise and oxytocin might interact with metabolic processes in WT and P2Y1 KO mice (Laplante et al., 2010).

WK and SHR rats showed that exercise activates oxytocinergic projections in their nucleus tractus solitarii, influencing oxytocin mRNA expression in the brain (Martins et al., 2005). The interplay between exercise metabolism and hormonal regulation, including the relationship between GLUT4 mRNA (Bakos et al., 2007) and oxytocin mRNA, suggests a potential indirect interaction between exercise-induced metabolic changes and oxytocin regulation in SD rats, the exploration of which could be useful in optimizing athletes’ training and recovery. Another study on W rats demonstrated acute exercise’s impact on oxytocin and cholecystokinin levels, along with their role in modulating gastric functions, highlighting the intricate neurohumoral mechanisms that are influenced by physical activity (Tolentino Bento da Silva et al., 2021). Similarly, another study examined stress induction and oxytocin administration in W rats connected to metabolism. Heat Shock Protein, the Hsp27 gene was significantly upregulated (Moghlmian et al., 2014), suggesting a link between oxytocin signaling and stress-related gene activation. W and SHR rats have been shown to increase oxytocin mRNA and immunoreactivity within key brain areas impacting autonomic control (Cruz et al., 2013), which might lead in principle to performance increase for human athletes later. Research on the effect of exercise on cardiovascular regulation in rats, focusing on phenylethanolamine N-methyltransferase (PNMT) gene expression and oxytocin’s cardiovascular response, has revealed significant exercise-induced changes in gene expression. In rats, voluntary wheel running failed to modify sensitivity to the cardiovascular action of oxytocin but resulted in increased gene expression of the PNMT gene in the left, but not right, heart atrium in a running activity-dependent manner (Bakos et al., 2008). In conclusion, the relationship between oxytocin, exercise, and genetics in animals appears to vary depending on the species. Nevertheless, the social aspect surrounding animals and their dietary attributes may be influenced by genetic underpinnings related to oxytocin. Further investigations could explore the presence and mechanisms of such changes in humans.

In humans, similar patterns can be perceived. A study linked the *SIM*1 gene to obesity, announcing that genetic factors influence body weight regulation (Swarbrick et al., 2011), which is parallel to animal studies also mentioning dietary variables (see chapter on nutrition). *AVPR1a* (central AVP receptor 1A) alongside oxytocin and higher cognitive levels can affect social variables of trust, and reciprocity (Nishina et al., 2019), which might contribute to understanding performance in team sports.

The most well-researched oxytocin receptor variant is *OXTR* rs53576 on human chromosome 3p.25.3. A meta-analysis on *OXTR* rs53576 polymorphism found a significant association with empathy, particularly in individuals with the GG genotype (Gong et al., 2017). Variations in *OXTR* rs53576 are associated with differences in trust behavior alongside AVPR1a. This finding indicates that oxytocin acts on trust-related elements whereas AVP acts on aspects linked to wider pro-social behavior and may address many forms of anxiety (Nishina et al., 2019). Genetic variation in *OXTR* rs53576 between cultures, linking it to differences in altruistic, empathic, and compassionate behaviors. In European Americans, the guanine variant (G) is more prevalent, but in collectivist East Asians, adenine (A) is more common. Carriers of *OXTR* rs53576 and *AVPR1a* are more likely to engage in prosocial behaviors, such as trust in strangers and participation in charitable activities, but an introduction of intranasal oxytocin showed varied results (Sonne and Gash, 2018). Interestingly, a study done in Japan showed that physical activity levels might interact with genetic predispositions to influence emotional and cognitive processes relevant to sports performance, particularly in individuals carrying the G variant of the *OXTR* rs53576 and *AVP V1b* (Shima et al., 2022). Lastly, *OXTR* rs53576 can even influence how empathetically one reacts to the pain of others outside or inside their racial group suggesting that there might be individual differences in oxytocin receptor functioning that can affect social and emotional processing (Luo et al., 2015).

In individual sports, the presence of *OXTR* rs53576 might not be as crucial as in team sports. Enhanced attention to empathy and trust, however, is crucial in the latter. It might be possible to test for these genes in the future so that people could choose appropriate sports, or sports and teams may choose optimal athletes (Sezen-Balçikanli and Sezen, 2017; Kavussanu and Stanger, 2017). A comparative study on the composition of humans participating in individual and team sports associated with their oxytocin levels would in all probability shed light on the interplay of genes in sports connected to oxytocin.

### 4.5 Social context

#### 4.5.1 Oxytocin as a reward related to sports in social context

Oxytocin and social context are closely related, the former being released as a reward connected to prosocial behavior in most vertebrate species (Wei et al., 2015; Glasper et al., 2012; Baran et al., 2016). Oxytocin is significantly correlated with perceived social support, playing a role in stress regulation, and promoting faster recovery (Pressman et al., 2013; Heinrichs et al., 2003). Positive verbal and tactile interactions facilitated the secretion of oxytocin in both humans and animals (Rassovsky et al., 2019; Nagasawa et al., 2022). The absence of social peers in rats increases the chances of cocaine addiction. Oxytocin lowered both the desired quantity of cocaine and the motivation to obtain it while reducing relapse behavior (Bentzley et al., 2014). Consequently, oxytocin might be instrumental in sports, to help athletes with motivation and potentially mitigate the risk of developing unhealthy or addictive behaviors. Stress-induced cortisol release can be buffered by positive social experiences and at least partially mediated by oxytocin release (Tawfik et al., 2023). In Tsimane’ hunters’ salivary oxytocin levels were significantly higher upon returning home compared to baseline, suggesting that oxytocin responds to social reunions and is likely involved in reinforcing social bonds and positive social interactions. Correlation between oxytocin levels and factors like meat sharing, cooperative hunting, family size, and physical activity indicate that oxytocin’s increase was specifically related to the social aspect of returning home (Jaeggi et al., 2015). A study on dogs and wolves found that synchronized movement was positively related to oxytocin concentrations. Strikingly, synchronized movements and singing (akin to howling perhaps) are present in humans, connected to oxytocin (Pfordresher, 2021). Territorial behavior was positively related to oxytocin and glucocorticoid release, and separation from the pack increased glucocorticoid concentrations, indicating that social and environmental factors, such as returning home or social reunions, can differently affect oxytocin levels in animals too (Wirobski et al., 2023). Consequently, it is suggested that the level of oxytocin should be measured at different time points (Fitzpatrick and Morrow, 2020).

#### 4.5.2 Fear connected to oxytocin and sports

The mechanism by which oxytocin promotes trust and alleviates social stress appears to involve deactivating brain circuits in the amygdala in both humans and rodents. The amygdala is involved in fear processing as a reaction to a fearful face in humans (Lv et al., 2022; Wang et al., 2017; Nishina et al., 2019; Frye et al., 2014; Murphy et al., 2012; Hahn et al., 2015) which is related to the mirror neuron system and empathy. On the other hand, in C57BL/6N mice oxytocin may enhance curiosity or interest in the environmental elements rather than directly promoting empathetic behavior, related to the transmission of fear (Ueno et al., 2019). In 129S1/SvImJ mice, this connection seems to be dose-dependent with even relatively low doses being sufficient to elicit positive behavior change and fear extinction memory (Gunduz-Cinar et al., 2020). Admittedly, further investigation is needed to interpret the last two findings for humans. In addition, music could be particularly effective in stimulating oxytocin release, thereby enhancing team cohesion and performance. It is relatively well-known that singing increases social bonding. This aligns with the idea that group activities, be they musical or athletic, serve an important role in social bonding facilitated by oxytocin (Pfordresher, 2021). Oxytocin can reduce social stress, and performance anxiety in musicians, further reducing negative or even catastrophic thoughts on professional activities. In a related study, oxytocin improved self-perception and helped to manage negative thoughts associated with music performance anxiety (Osório et al., 2022). This is in line with the finding that athletes who perceive high social support, such as from coaches, teammates, or fans, might have higher oxytocin levels, facilitating a challenge state and enabling them to utilize favorable conditions effectively, thereby maximizing their performance potential (Meijen et al., 2020). Oxytocin may be increased by stress such as pain, fear, and intense exercise in rats, humans, and horses. A study found it was exercise that facilitated meaningful oxytocin-level change in horses (Kim et al., 2023; Hada et al., 2003). Furthermore, horses with a high concentration of oxytocin show lower levels of fearfulness and dominant behavior, which are associated with better trainability (Kim et al., 2021).

#### 4.5.3 Oxytocin as the trust hormone in sports

A crucial function of oxytocin is the early processing of the most basic class of social stimuli which is most often the face (Fujimura and Okanoya, 2016; Rimmele et al., 2009). Social cognition, recognition, and memory are essential prerequisites of more complex social behaviors both of which are enhanced and partly regulated by oxytocin (Zeng et al., 2012; Applebaum et al., 2023; Atsumi et al., 2018; Squires et al., 2006; Kis et al., 2015; Rimmele et al., 2009). Athletes' ability to remember and learn from social interactions and team experiences also could be shaped by hippocampal mechanisms connected to oxytocin (Stöber et al., 2020; Lehr et al., 2021). Subsequently, locomotive activity and oxytocin may be connected. Compared to healthy controls, CD157 and CD38 KO mice (oxytocin release is disturbed) showed decreased locomotor activity and increased immobility, along with an inability to differentiate between familiar and novel social stimuli (Gerasimenko et al., 2020). Oxytocin can be looked at as a double-edged sword in relation to sports teams. While it promotes selective trust inside groups, on some level it facilitates negative attitudes towards others outside the given group (Hattori et al., 2015; Bershad et al., 2016; Landon et al., 2018). Oxytocin is capable of amplifying both positive and negative social interactions (Bernaerts et al., 2016; Bellosta-Batalla et al., 2020; Peen et al., 2021; Leeds et al., 2020; Steinman et al., 2019). The dynamic nature of trust, as indicated by changes in trust levels during trust games, could also parallel how exercise-induced physiological changes (like fluctuations in oxytocin levels) may influence social behaviors and trust in a sports setting, even synchronize heart rates and arousal (Hahn et al., 2015; Mitkidis et al., 2015). Oxytocin was found to lower the moral acceptability of outcome-maximizing choices, which might take part in moral decision-making (Palumbo et al., 2020) of athletes indirectly, leading to a more humanistic behavior in sports through empathy. As of yet, the aforementioned findings are genetic profile-related (Gong et al., 2017; Sonne and Gash, 2018). Interestingly, excess exogenous oxytocin might have other effects on outsiders from the group. Oxytocin even increased participants’ willingness to trust strangers but did not change their willingness to take non-social risks (Wang et al., 2017) which might be meaningful in eliciting team cohesion. By the same token, individuals who received oxytocin did not decrease their trust in response to a partner’s breach of trust, while those participants who had received a placebo adjusted their behavior naturally.

#### 4.5.4 Play, physical touch, and oxytocin in sports

Play and eliciting playful behavior in both animals and humans show a significant link to oxytocin levels, particularly in social contexts. This is evident in studies examining interactions between pets and their owners, as well as father-infant dynamics, highlighting oxytocin’s role in enhancing social bonding and trust, potentially influencing sports performance (Mitkidis et al., 2015; Gray et al., 2017; Nagasawa et al., 2020; Rossi et al., 2018; Schreiner, 2016). Oxytocin’s role in sports-related physical contact underlines its significance in enhancing team cohesion, trust, and overall wellbeing in athletes (Mitsui et al., 2011; Kociuba et al., 2023; Frey Law et al., 2008; Bellosta-Batalla et al., 2020; McGuire et al., 2015; Rossi et al., 2018). Physical touch in sports, such as grappling, instead of kicking and punching can lead to increased oxytocin levels, enhancing social bonding and reducing stress (Rassovsky et al., 2019; Haynes et al., 2022) hinting at the idea, that the intensity and salience of the touch might matter. Both human and animal studies support that, oxytocin release during exercise, depending on physical contact may contribute to positive emotional responses and improved social interactions among athletes (Leggieri et al., 2019; Niittynen et al., 2022; Damian et al., 2022; Baran et al., 2016; Cavalli et al., 2018; Mengoli et al., 2021; Nagasawa et al., 2020). Furthermore, these positive effects of oxytocin, released during physical contact in sports, can extend beyond the activity itself, influencing mood and social behaviors (Behm et al., 2020; Haynes et al., 2022; Limanowski, 2022).

Oxytocin is influenced by social interactions, and *vice versa*. Team bonding and physical contact, alongside playful behaviors, are common in sports settings. The type of sport therefore seems a determining factor regarding the aforementioned variables. Consequently, oxytocin’s influence on athletes’ social behavior and its performance-enhancing effect proposes interesting moral dilemmas about this neuropeptide as it might have potential uses for pro-athletes both increasing performance and recovery. Future studies could delve into dose-response reactions in humans regarding fear, performance, and trust. Regarding psychometric testing with the Multidimensional Scale of Perceived Social Context (MSPSS) individuals reporting very low levels could potentially be excluded from the samples.

### 4.6 Substances and nutrition

The relationship between AVP and oxytocin is complex, and the exact mechanisms aren't fully understood yet. Factors like individual variability, overall health, and hormonal feedback loops can all influence this interaction. Even still, both animal and human studies prove that water and salt intake may influence blood pressure, AVP, and consequently oxytocin (Michelini, 2007a; Michelini, 2007b; Yokoyama et al., 2009; Duale et al., 2007; Kawasaki A. et al., 2005; Kawasaki M. et al., 2005; Kozoriz et al., 2006). When AVP levels rise, it can affect the release of oxytocin because they share common pathways and receptors (Kawasaki A. et al., 2005; Bhumbra et al., 2008; Nakamura et al., 2011). This is possible as an example, through the endocannabinoid system (Ruginsk et al., 2010) Improved hydration strategies for athletes with water-salt intake connected to oxytocin may lead to meaningful results in recovery, through cardioprotection and pain management.

Food intake behavior is affected by oxytocin, lowering appetite. Oxytocin could offer heart protection from dietary problems, help the regeneration of multiple tissues, and both directly and indirectly reduce obesity, through portion control both in humans [in the hedonic (pleasure-related) and homeostatic (energy balance-related) aspects of feeding] and animals (Espinoza et al., 2021; Mitsui et al., 2011; Yokoyama et al., 2009; Anekonda et al., 2021; Head et al., 2019; Nakamura et al., 2011; Gosnell and Levine, 2009; Lohmeier et al., 2003; Shimizu et al., 2016; Skinner et al., 2019; Yao et al., 2022). Admittedly, this needs additional research on humans, particularly dose-response studies, and an understanding of related genetics (Swarbrick et al., 2011).

In Zucker fatty (ZF) rats, circulating oxytocin levels were accompanied by significantly improved glucose utilization, highlighting the potential role of oxytocin in metabolic regulation, which could be influenced by nutritional status (Krskova et al., 2020). A study examined neuromedin U, which facilitates oxytocin release, at least in part, via activation of beta-adrenoceptors in W rats (Rokkaku et al., 2003), acting similarly on dietary intake related to oxytocin. Ghrelin has been used as an antagonist for inducing hunger by reducing oxytocin (Yokoyama et al., 2009; Szabó et al., 2019). At the same time, oxytocin was found to inhibit the increase in neuropeptide Y levels induced by ghrelin in humans (Coiro et al., 2008), leading to inconclusive evidence in light of the aforementioned studies for now. Aromatherapy through essential oils may hold promise for assisting athletes’ dietary changes through increased oxytocin release (Cai et al., 2023). Alcohol and painkillers may decrease oxytocin, potentially slowing recovery processes (Jhamandas and MacTavish, 2003; Stevenson et al., 2017; Kandis et al., 2018; McGuire et al., 2015; Skinner et al., 2019). Centrally administered oxytocin can reduce the feeling of visceral gastrointestinal sensations in SD rats, pointing out its possible involvement in pain relief. If this is confirmed in humans, dietary components might influence central oxytocin levels and, consequently, pain perception and stress responses (Okumura et al., 2019). In addition to this, circadian rhythms may influence the measurements through dietary and hormonal, neural pathways, possibly influencing exercise performance (Bhumbra et al., 2009; Sugiyama et al., 2020). Oxytocin’s role in nutrition concerning sports science is a developing area of research, that holds promising potential for increased performance and lowered recovery times through several biomarkers being influenced in both humans and animals (Lesimple et al., 2020; Zhuang et al., 2021; Laplante et al., 2010; Beckner et al., 2021). Attention is needed for proper pharmaceutical solutions to explore and utilize these findings, which could mean psychophysiological improvements for all humans (Ohno et al., 2009).

It is crucial to consider the fluid, salt intake, and diets athletes consume as this may be a determining factor in oxytocin release. Examining the correlation between pain and cognitive functions alongside other factors mentioned in this review might provide insight into how this hormone works exactly. The Council of Nutrition Appetite Questionnaire (CNAQ) (Hanisah et al., 2012) or Food Frequency Questionnaire (FFQ) (Shahar et al., 2003) as an alternative, for nutrition encompasses a great variety of nutritional variables for this purpose. Furthermore, the ethical considerations here are crucial, meaning the development of drugs and therapies must be carefully undertaken. It is possible that athletes have used oxytocin to boost performance and recovery for a time, and because of the half-life of this neuropeptide in the body, this must have been difficult to test for, if not impossible.

### 4.7 Stress

#### 4.7.1 Oxytocin’s role in stress reduction

Oxytocin is often used in both human and animal studies as a biomarker of positive health status (Indrayan et al., 2023; Lerner et al., 2022). When under stress caused by exercise, W rats produce the adrenocorticotropic hormone, in response to which oxytocin is also secreted as a buffer in the stress response. However, in humans, this might be dependent on the stress scenario, the environment, genetic profiles, early life experiences, and nutrition (Takahashi et al., 2022; Liu et al., 2022; Scarola et al., 2019; Skinner et al., 2019; Cai et al., 2023; Ebner et al., 2005; Noto et al., 2021; Weisman et al., 2013). At the same time, individual variations will contribute to how much stress gets reduced by oxytocin (Fitzpatrick and Morrow, 2020; Sonne and Gash, 2018). Prolonged endurance exercise, a form of physical stress, triggers significant hormonal changes, including increases in oxytocin levels significantly correlating with AVP, suggesting that exercise can modulate stress responses through hormonal pathways (Hew-Butler et al., 2008a; Hew-Butler et al., 2008b). Oxytocin seems to inhibit social stress and attenuate activation of the amygdala response to a fearful face, making faces, more familiar” (Rimmele et al., 2009). Oxytocin and AVP may have opposite effects on the brain in response to social stress and cardiovascular control (Gutkowska et al., 2016; Crestani et al., 2013; Nishina et al., 2019), which might not be a coincidence. Acute osmotic change as physiological stress may alter a response in oxytocin because of the relationship between the two hormones (Kawasaki A. et al., 2005; Kawasaki M. et al., 2005). Both chronic and acute stress experiences can significantly alter neuroendocrine responses, specifically involving oxytocin and corticotropin-releasing hormone and their interaction (Peen et al., 2021), suggesting that social interactions play a crucial role in modulating these stress responses both in animals and humans (Pressman et al., 2013; Lv et al., 2022; Gerasimenko et al., 2020; Frye et al., 2014; Leeds et al., 2020; Trieu et al., 2019). In addition, the endocannabinoid system takes part in the regulation of social reward by oxytocin reinforcing the connection with social context further (Wei et al., 2015). In sports contexts where team dynamics and social support are key factors, both the variables are important.

#### 4.7.2 Acute stress

In humans, acute stress impacts cognitive functions and the potential buffering effects of oxytocin. Interestingly, oxytocin which is related to resilience and fitness, remained stable during simulated military operational stress, although the study notes problems with oxytocin measurement unreliability (Beckner et al., 2021), which this current review aims to address directly. With regards to acute stress, the immediate connection between heart rate and cardioprotection, with hormonal changes may contain valuable information about oxytocin’s role in the stress response (Moghimian et al., 2012; Moghlmian et al., 2014; Dampney and Horiuchi, 2003; Yao et al., 2022) offers promising research areas for the future.

Athletes, with varying levels of social risk-taking tendencies, might respond to acute stressors in competitive sports differently (Wang et al., 2017). Individuals’ propensity for social risk-taking influences their trust decisions in uncertain social interactions (Bershad et al., 2016).

Exercise, by affecting oxytocin levels, might be a valuable component in managing stress and improving mental health in various populations, including those with addiction issues (Wang and Zou, 2022). Likely through the opioid receptors in the mPFC, forced swimming as acute stress and its impact on meth and morphine-seeking behavior in humans and W rats suggests that stress can significantly influence neurochemical activity related to reward and motivation (Farzinpour et al., 2019; Carson et al., 2010; Kim and Han, 2016; Salighedar et al., 2019). Similarly, the MWM test exposure also triggered a transient release of oxytocin (Engelmann et al., 2006). Interestingly, even with music playing backward as an acute stressor, concentrations of testosterone, oxytocin, AVP, and aldosterone were slightly increased accompanied by increased anxiety (Jezova et al., 2013).

The amygdala’s role in fear extinction, as observed in male S1 mice, provides insights into how stress affects athletes' neurochemical pathways during competition. This is similar to studies on C57BL/6J mice and oxytocin receptor-deficient mice, where oxytocin receptors are linked to behavioral responses to social stress. In human athletes facing competitive stress, the modulation of emotional and stress responses by oxytocin suggests a parallel mechanism. Oxytocin may influence stress and fear-related processes in athletes, mirroring the neurochemical pathways activated in these mice models during stress.

Furthermore, oxytocin predicts behavioral responses to foundation training in horses as an acute stress scenario, where cortisol is continually reduced for 9 months likely due to oxytocin. Training in horses can lead to measurable changes in oxytocin levels, which are associated with different behavioral responses (Niittynen et al., 2022). This suggests that in sports contexts, acute stress situations might similarly influence oxytocin levels, helping with acclimatization periods to training.

#### 4.7.3 Chronic stress

Oxytocin has been shown to play an important role in wound healing via modulating stress responses by activating the processes of neovascularization, and proliferation of endotheliocytes and histiocytes (Gümüs et al., 2015). This may result in effective clearance of the wound, and optimal granulation tissue formation, which may also help athletes if tested on humans later in their recovery processes. Furthermore, chronic exercise training can modulate oxytocinergic and vasopressinergic pathways, affecting cardiovascular control. The long-term impact of regular exercise on these neuroendocrine systems may influence stress responses in athletes (Michelini, 2007a; Michelini, 2007b). Chronic stress boosts the levels of certain hormones like orexin, melanin-concentrating hormone, oxytocin, AVP, and thyrotropin-releasing hormone in the basolateral amygdala. On the other hand, exercise has an antidepressant effect by reducing the expression of these hormones in the basolateral amygdala, further strengthening oxytocin’s critical role in mood regulation (Kim and Han, 2016). A study examined the effects of continual hypoxia, which is a form of chronic stress, on hormone regulation. C57BL/6 mice were subjected to chronic stress (restraint treatment) and moderate physical exercise (running wheel). Acute continual hypoxia-affected prolactin levels, mediated through corticotropin-releasing hormone receptor 1, may affect oxytocin levels in SD rats (Dampney and Horiuchi, 2003). Acute hypoxia can be caused by anemia or asthma, training swimming, or chronic stress in athletes.

Vasopressin’s response to chronic stress could imply that similar mechanisms may influence oxytocin levels under chronic stress in athletes. Understanding how chronic stressors, like intensive training or injury, might impact athletes’ neuroendocrine responses and, subsequently, their performance and recovery processes are likely connected to the regulation of AVP (Suzuki et al., 2009). Sustained inflammatory stress on AVP-eGFP Wistar transgenic rats, was used to analyze the expression of an AVP-enhanced green fluorescent protein fusion gene in response to chronic inflammatory stress. Furthermore, chronic corticosterone administration as a model of chronic stress in W rats suggests, that oxytocin may have protective effects against stress-induced changes in the adrenal gland (Stanić et al., 2017) as one potential theory of how exercise may promote stress reduction through oxytocin. In prairie voles, the influence of social bonds and their disruption on both physical (pain perception) and psychological (anxiety) aspects of social stress due to partner loss could significantly impact how individuals respond to stressors connected to oxytocin (Osako et al., 2018), which needs to be accounted for in the measurements for humans.

The type of stress may be a determining factor in the secretion of oxytocin due to exercise. Stress management for athletes is a crucial step for sports science and psychology. To know more about what kind of stress affects oxytocin, and how this interacts with other psychological and physiological systems documenting individual results with the Perceived Stress Scale (PSS) alongside the Numerical Rating Scale (NRS) may be informative to notice important correlations. The type of exercise will definitely play a role in the amount of stress experienced by athletes, and their response to said stress, because of the endogenous oxytocin released.

### 4.8 Limitations of the evidence included in the review

While this review incorporates frequent animal studies, they do not always have direct applicability to humans. However, it was deemed necessary for maintaining the stability of the measurement framework. The English language restriction could also limit the review’s scope. Furthermore, the reviewer processes, involving independent work and subsequent online debates, may introduce potential biases. Despite these limitations, this review aims to establish a robust methodology framework for measuring a neuropeptide and exploring correlation studies, contributing to the understanding of the complex interplay between exercise and oxytocin. Utilization of a business intelligence tool with automation, like Power BI for thematic analysis and statistical validation may also offer insights for future methodological studies.

#### 4.8.1 Limitations of the review processes used

At some points, the two reviewers worked independently, and the debates were held later online in meetings, the results of which were documented in the Excel sheets, with reviewer notes and data validation following each decision.

#### 4.8.2 Implications of the results for practice, policy, and future research

Our review aims to build a methodology framework for measuring a neuropeptide and finding possible tests for important correlation studies. Furthermore, it builds an understanding of exercise and oxytocin’s interplay. Utilizing Power BI for thematical analysis through data validation and statistics may lead to new methodological studies and frameworks. Our findings may be directly and indirectly applicable to sports and medical science.

## Data Availability

The original contributions presented in the study are included in the article/Supplementary Material, further inquiries can be directed to the corresponding author.

## Abbreviations

AVP, Arginine vasopressin; KO mice, knockout mice; LE rat, long-evans rat; mPFC, medial prefrontal cortex; MWM, morris water maze; OXTR, oxytocin receptor gene; SD rat, sprague dawley rat; SHR rat, spontaneously hypertensive rat; WKY rat, Wistar Kyoto rat; W rat, wistar rat; ZF rat, zucker fatty rat.
