# Supplementary material for: Systematic review: pain, cognition, and cardioprotection—unpacking oxytocin’s contributions in a sport context
Source: Front Physiol. 2024 Jun 10;15:1393497. doi: 10.3389/fphys.2024.1393497 (PMC11194439; doi:10.3389/fphys.2024.1393497)
Supplement: Supplementary file 1 [file Presentation1.PDF]

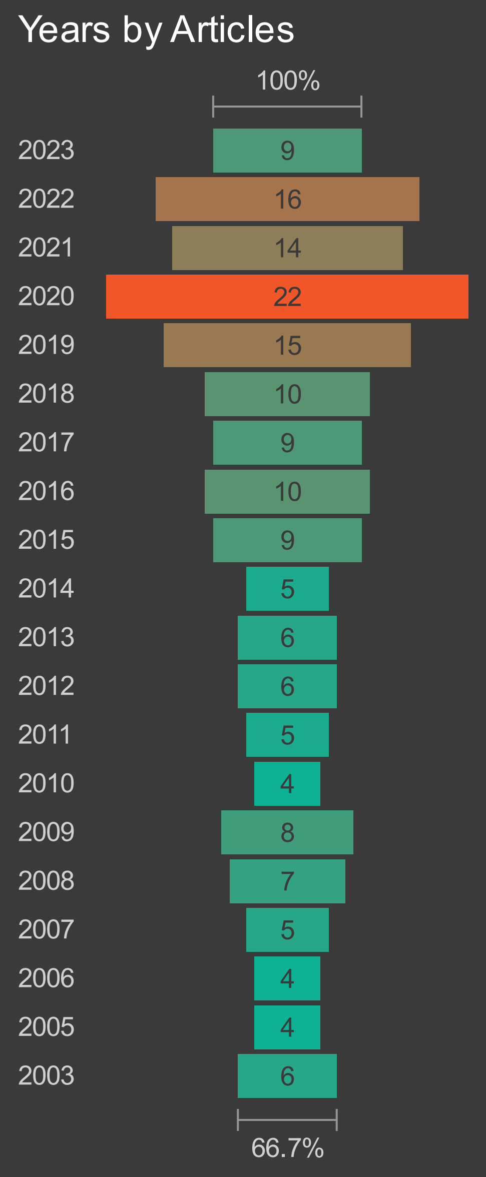
Years by Articles
100%
2023
9
2022
16
2021
14
2020
22
2019
15
2018
10
2017
9
2016
10
2015
9
2014
5
2013
6
2012
6
2011
5
2010
4
2009
8
2008
7
2007
5
2006
4
2005
4
2003
6
66.7%

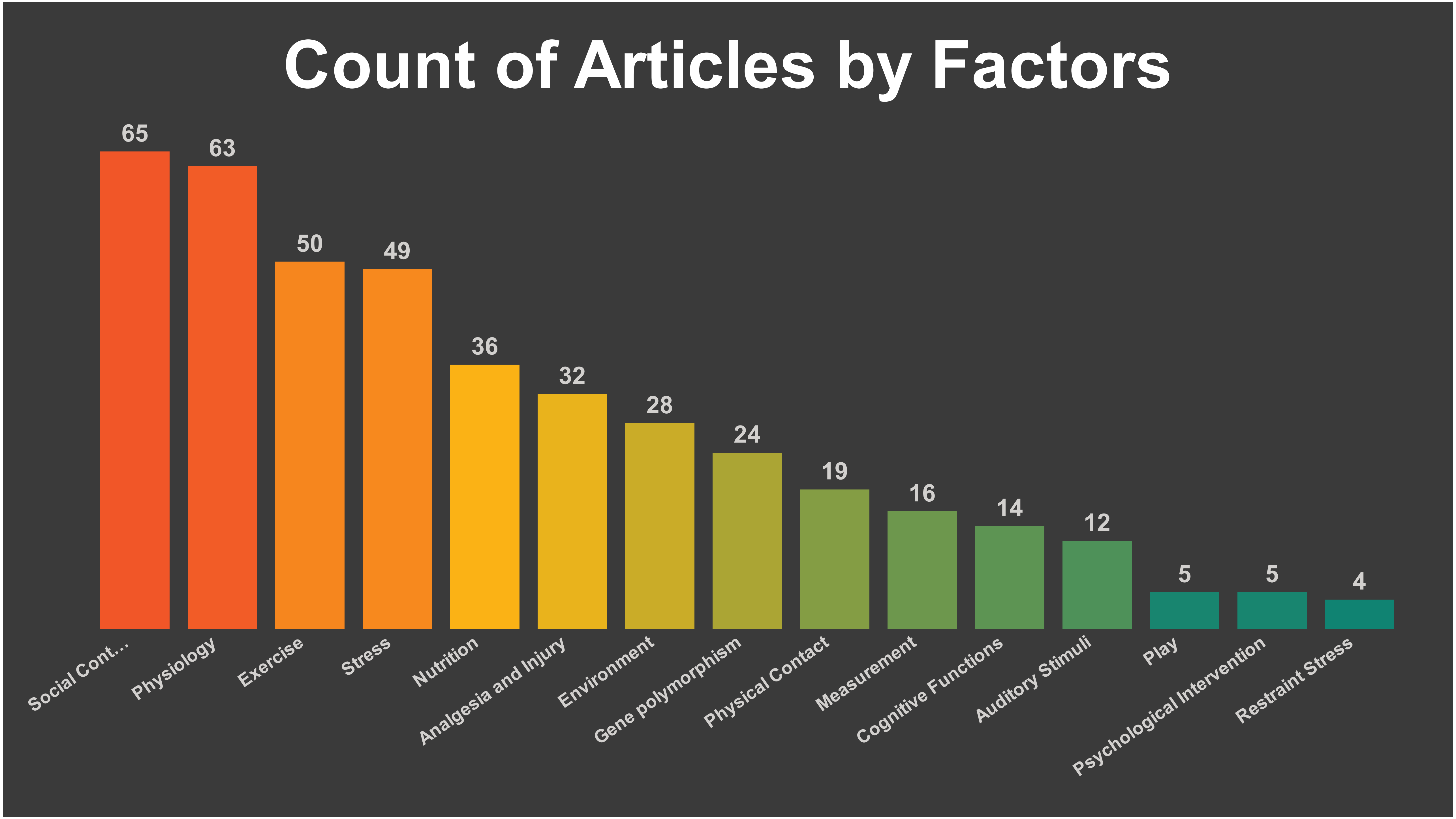
Count of Articles by Factors
65
63
50
49
36
32
28
24
19
16
14
12
5
5
4
Social Cont...
Physiology
Exercise
Stress
Nutrition
Analgesia and Injury
Environment
Gene polymorphism
Physical Contact
Measurement
Cognitive Functions
Auditory Stimuli
Play
Psychological Intervention
Restraint Stress

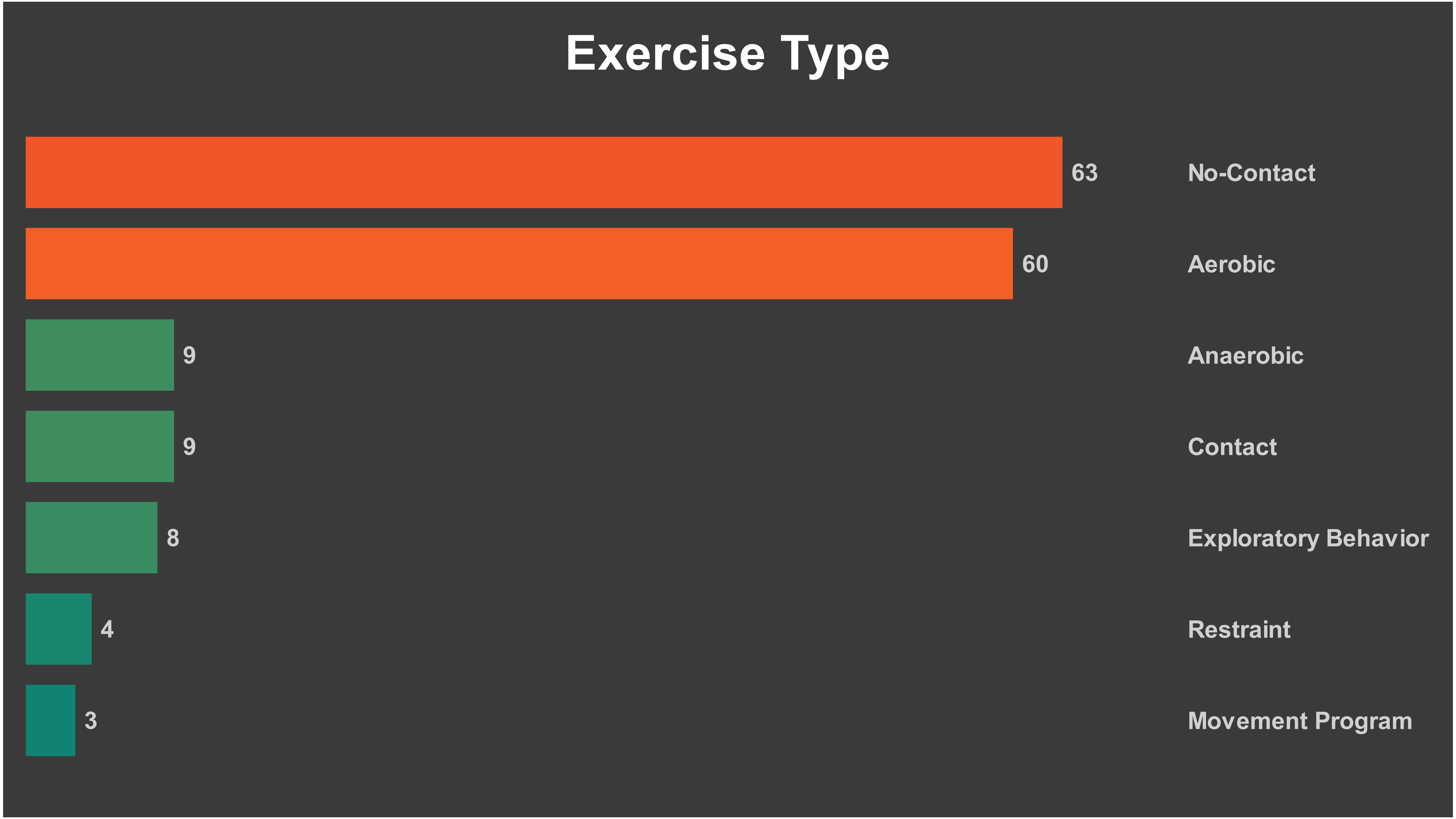
Exercise Type
63
No-Contact
60
Aerobic
9
Anaerobic
9
Contact
8
Exploratory Behavior
4
Restraint
3
Movement Program

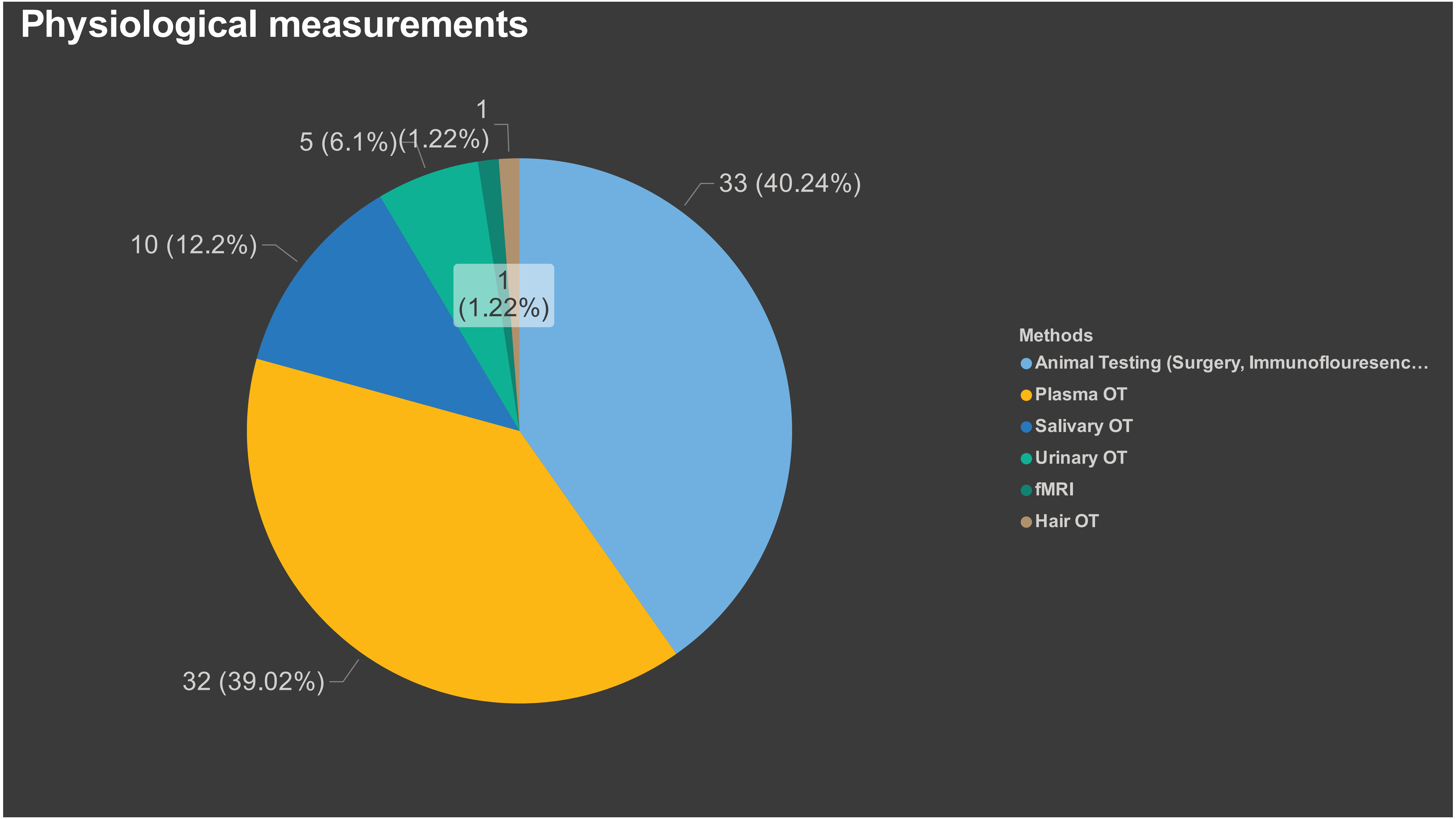
Physiological measurements
1 (1.22%)
5 (6.1%)
33 (40.24%)
10 (12.2%)
1 (1.22%)
32 (39.02%)
Methods
Animal Testing (Surgery, Immunoflouresenc...
Plasma OT
Salivary OT
Urinary OT
fMRI
Hair OT

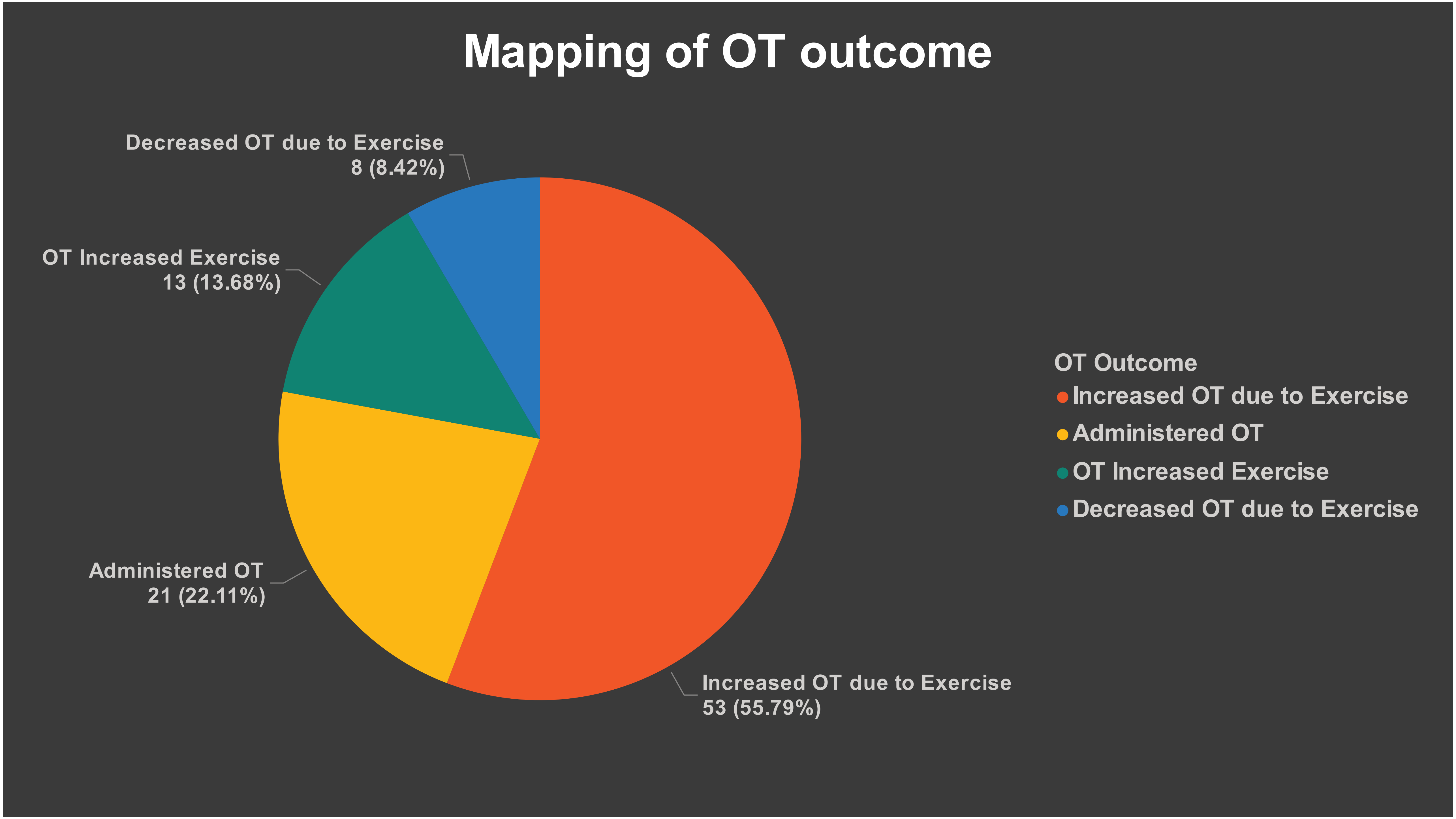
Mapping of OT outcome
Decreased OT due to Exercise
8 (8.42%)
OT Increased Exercise
13 (13.68%)
Administered OT
21 (22.11%)
Increased OT due to Exercise
53 (55.79%)
OT Outcome
Increased OT due to Exercise
Administered OT
OT Increased Exercise
Decreased OT due to Exercise

RoB Assessment

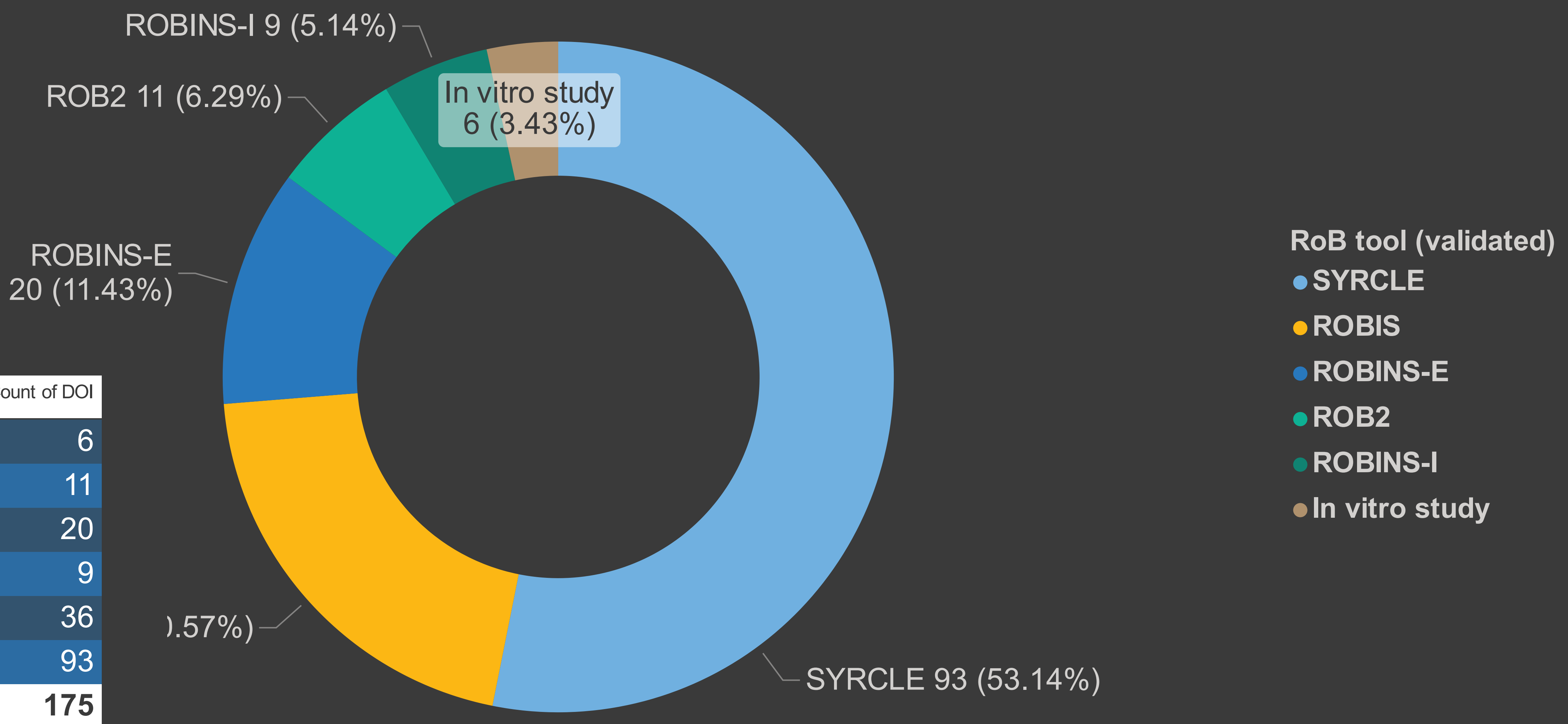

RoB tool (validated)	Count of DOI
In vitro study	6
ROB2	11
ROBINS-E	20
ROBINS-I	9
ROBIS	36
SYRCLE	93
**Total**	**175**

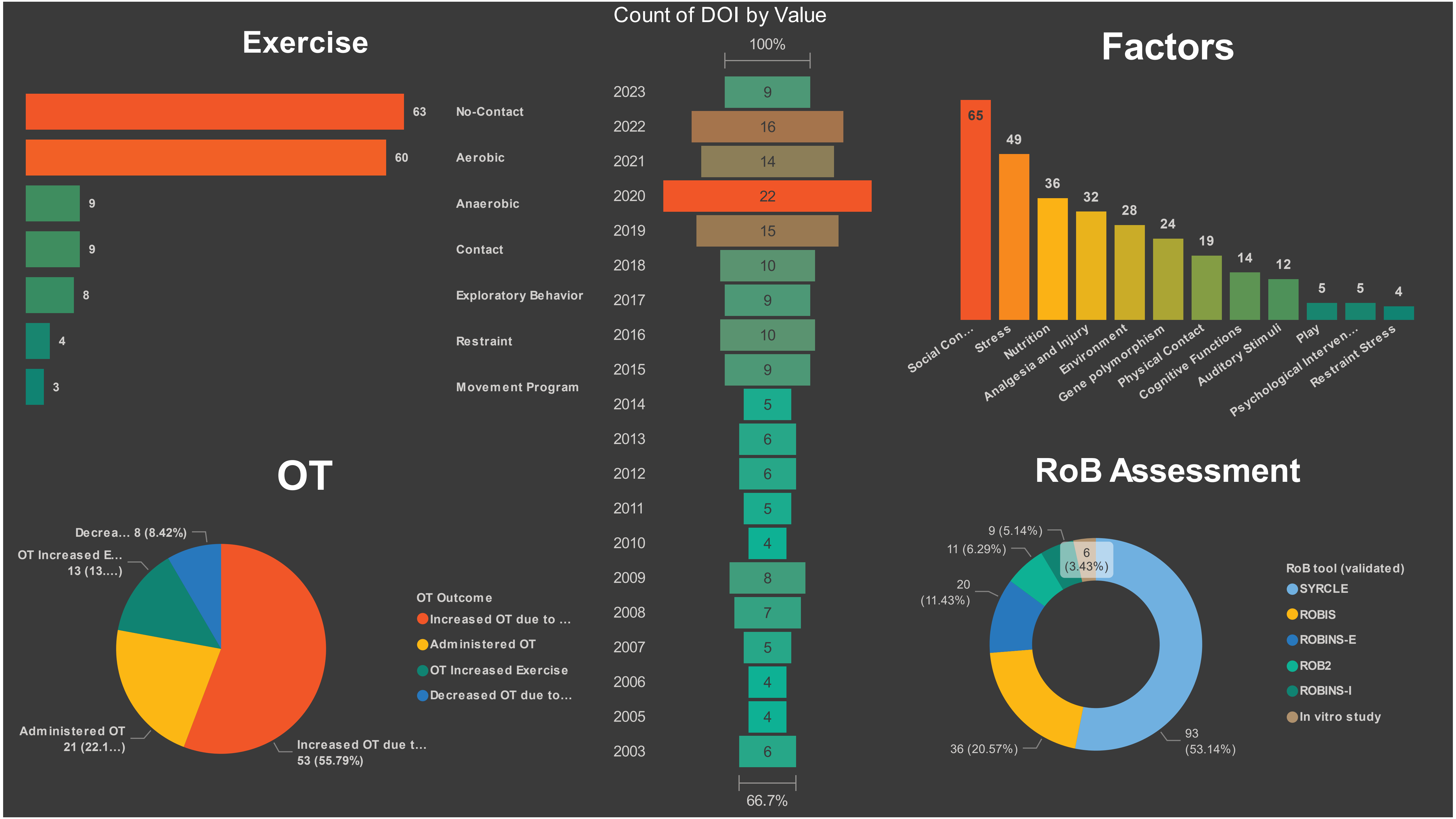
Exercise
63 No-Contact
60 Aerobic
9 Anaerobic
9 Contact
8 Exploratory Behavior
4 Restraint
3 Movement Program
Count of DOI by Value
100%
2023 9
2022 16
2021 14
2020 22
2019 15
2018 10
2017 9
2016 10
2015 9
2014 5
2013 6
2012 6
2011 5
2010 4
2009 8
2008 7
2007 5
2006 4
2005 4
2003 6
66.7%
Factors
65 Social Con...
49 Stress
36 Nutrition
32 Analgesia and Injury
28 Environment
24 Gene polymorphism
19 Physical Contact
14 Cognitive Functions
12 Auditory Stimuli
5 Play
5 Psychological Interven...
4 Restraint Stress
OT
Decrea... 8 (8.42%)
OT Increased E... 13 (13....)
Administered OT 21 (22.1...)
Increased OT due t... 53 (55.79%)
OT Outcome
Increased OT due to ...
Administered OT
OT Increased Exercise
Decreased OT due to...
RoB Assessment
9 (5.14%)
11 (6.29%)
6 (3.43%)
20 (11.43%)
36 (20.57%)
93 (53.14%)
RoB tool (validated)
SYRCLE
ROBIS
ROBINS-E
ROB2
ROBINS-I
In vitro study
